# Online Prevention Aimed at Lifestyle Behaviors: A Systematic Review of Reviews

**DOI:** 10.2196/jmir.2665

**Published:** 2013-07-16

**Authors:** Leonie FM Kohl, Rik Crutzen, Nanne K de Vries

**Affiliations:** ^1^Department of Health PromotionCAPHRI School for Public Health and Primary CareMaastricht UniversityMaastrichtNetherlands

**Keywords:** eHealth, telemedicine, Internet, prevention, life style, health behavior, RE-AIM

## Abstract

**Background:**

Interventions aimed at behavior change are increasingly being delivered over the Internet. Although research on intervention effectiveness has been widely conducted, their true public health impact as indicated by reach, effectiveness, and use is unclear.

**Objective:**

The aim of this paper is to (1) review the current literature on online prevention aimed at lifestyle behaviors, and (2) identify research gaps regarding reach, effectiveness, and use.

**Methods:**

A systematic search in PubMed revealed relevant literature published between 2005 and 2012 on Internet-delivered behavior change interventions aimed at dietary behaviors, physical activity, alcohol use, smoking, and condom use. Our search yielded 41 eligible reviews, which were analyzed in terms of reach, effectiveness, and use according to the RE-AIM framework.

**Results:**

According to health priorities, interventions are largely targeted at weight-related behaviors, such as physical activity and dietary behavior. Evaluations are predominantly effect-focused and overall effects are small, variable, and not sustainable. Determinants of effectiveness are unclear; effectiveness cannot yet be unambiguously attributed to isolated elements. Actual reach of interventions is undiversified, mostly reaching participants who are female, highly educated, white, and living in high-income countries. One of the most substantial problems in online prevention is the low use of the interventions, a phenomenon seen across all behavior domains.

**Conclusions:**

More research is needed on effective elements instead of effective interventions, with special attention to long-term effectiveness. The reach and use of interventions need more scientific input to increase the public health impact of Internet-delivered interventions.

## Introduction

Unhealthy lifestyles have a major impact on morbidity and mortality [1]. Health promotion is the process of enabling people to increase control over their health and its determinants, thereby improving their health [2]. This also entails interventions targeting lifestyle behaviors. Health promotion is shifting its gaze toward new delivery modes (eg, the Internet) to effectively reach a larger part of the population with interventions targeting lifestyle behaviors. Access to the Internet is growing, especially in high-income areas, such as the United States (78.6%) and Europe (63.2%) [3]. Therefore, Internet-delivered interventions have become a well-established instrument within the health promoter’s toolbox to potentially reach a large part of the population. Internet-delivered interventions can be operationalized as “typically behaviorally or cognitive behaviorally-based treatments that have been operationalized and transformed for delivery via the Internet”. Usually, they are highly structured; self or semi self-guided; based on effective face-to-face interventions; personalized to the user; interactive; enhanced by graphics, animations, audio, and possibly video; and tailored to provide follow-up and feedback” [4].

The advantages of using the Internet as a delivery mode for health promotion are numerous. From a users’ point of view, the Internet is accessible 24/7 and interventions can be used anonymously and at any pace [5]. Anonymity is favorable for interventions regarding behaviors that might involve shame (eg, condom use, alcohol moderation) [6]. Internet-delivered interventions may reach populations who otherwise would not receive treatment [7]. From a health promoters’ point of view, (1) the Internet offers improved opportunities for maintenance and updating of interventions [8], (2) Internet-delivered interventions can mimic expensive face-to-face sessions in providing an individual as well as interactive approach in combination with an increased reach [9,10], and (3) Internet-delivered interventions are adjustable relatively easily to the needs of the user [11]. The advantageous characteristics of Internet-delivered interventions are also related to the downsides. Anonymity and limited face-to-face contact, for instance, can lead to high attrition rates [12]. This might affect the public health impact of these interventions.

Still, Internet-delivered interventions seem to possess potential with respect to health behavior change [13], but the actual public health impact remains unknown. Within the field of public health, intervention research is predominantly focused on intervention effectiveness [14], or even on effective elements [15]. This research focus oversimplifies reality in the quest to identify efficacious interventions. Effectiveness alone may reflect internal validity, but many interventions that prove to be effective in trials are much less effective when disseminated outside the context of a trial [16]. It has become apparent that reach and use of interventions are at least as important as effectiveness, because the most effective intervention will not have a public health impact if its reach or actual use by the target group is low [17].

The RE-AIM framework acknowledges that reach, effectiveness, adoption, implementation, and maintenance (hence, the acronym RE-AIM) are factors that all contribute to the public health impact of an intervention [18]. Therefore, this study focuses on the public health impact of Internet-delivered interventions by taking all these factors into account [19]. In the RE-AIM framework, reach is described as the percentage of individuals affected by a policy or program, but also the characteristics of those individuals [19]. In this review, effectiveness is defined as changes in behavioral outcomes. Originally, the RE-AIM framework described adoption, implementation, and maintenance as factors at the organizational level. Internet-delivered interventions, however, can often be used standalone, which means that adoption, implementation, and maintenance also takes place (at least partly) at the individual level: each user decides whether to visit an intervention website for the first time (adoption), and whether to keep using it as intended (implementation), and for the long term (maintenance) [20,21]. These factors of the RE-AIM model are defined as *use* within the current study.

Within the field of public health, the use of the Internet as (the primary) delivery mode has expanded substantially and it is hard to imagine the public health field without the Internet. However, as mentioned previously, the public health impact as indicated by reach, effectiveness, and use remains unknown. This literature study comprises a systematic review of reviews addressing the following research questions:

1. What is the reach, effectiveness, and use of Internet-delivered interventions aimed at lifestyle behaviors (ie, dietary behaviors, physical activity, alcohol use, smoking, and condom use)?
2. What are the gaps in our current knowledge about the public health impact of Internet-delivered interventions aimed at lifestyle behaviors?

## Methods

### Search Strategy

We identified relevant publications by conducting a PubMed search. The search query was designed in a way that both a search word regarding one of the behaviors of interest was in the title or abstract as well as a search word indicating the use of the Internet as (primary) delivery mode. Terms (including spelling variations and synonyms) that we searched for were eHealth OR Internet AND physical activity (exercise, sport, exertion, training, energy balance), smoking (tobacco, cigarette), alcohol (drinking, AOD, substance), nutrition (food, eat, weight, obesity, overweight, diet, adiposity), sexuality (safe sex, condom, HIV, AIDS, STI, STD), OR behavior (health, lifestyle, prevention, intervention). The exact search query can be found in Multimedia Appendix 1.

The search was conducted in December 2012 and was limited to systematic reviews and meta-analyses published from 2005 to 2012 in the English language. Prior research was covered by a study conducted in 2005 by De Nooijer et al [22] in which no reviews were available.

### Selection Criteria

Systematic reviews and meta-analyses were included if they (1) described at least 2 primarily Internet-delivered interventions aimed at behavior change regarding physical activity, smoking, alcohol use, dietary behaviors, or condom use, (2) reported on reach, effectiveness, or use of the included interventions, (3) were aimed at primary or secondary prevention for (part of) the general population, and (4) were available (full text) in English. Interventions aimed at health care workers or other intermediates were excluded. The article selection as well as data extraction was for all studies independently performed by 2 researchers (LK and RC) employing a conservative approach. If 1 of the 2 researchers was in doubt based on the title, the article was taken to the next round assessing the abstract. If 1 of the 2 researchers was in doubt based on the abstract, the article was taken to the next round assessing the full text. Disagreements were discussed until full consensus was reached.

### Search Outcome

**Figure 1:**
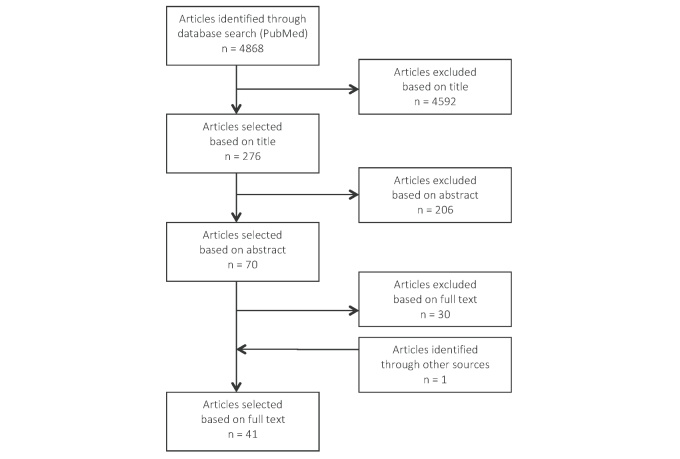
Flowchart of study selection process.

The database search resulted in 4868 articles. Initial review was based on the title, after which 276 articles remained. Based on abstracts, 206 articles were excluded leaving 70 articles for a full-text analysis. Reasons for exclusion of articles based on full text (n=30) were that the publication was not a systematic review or meta-analysis (10/30, 33%), contained less than 2 eligible interventions (9/30, 30%), did not report measures on reach, effectiveness, or use of the interventions reviewed (5/30, 17%), were not primarily Internet-delivered (4/30, 13%), were aimed at treatment of a disease (1/30, 3%), or the full text was not available in the English language (1/30, 3%). Some articles were excluded for more than 1 reason. One article was retrieved in a manual search. Reference lists of the selected articles were checked for possible missed publications, but yielded no additional articles. Forty-one articles were found to be eligible for this review (Figure 1).

An additional search was conducted in May 2013. This search extended the initial search by including the terms mhealth, smartphone, and mobile. In the initial search, these terms were not included because the focus of the study was on interventions that are primarily Internet-delivered. Therefore, reviews focusing on text messaging or apps were not included. Internet-delivered interventions are delivered by means of websites and it might indeed be that these are delivered by means of websites especially designed to be used/accessed on mobile phones. The additional search resulted in 174 hits (using the same limitations as the initial search). After title and abstract selection, there were 7 articles appropriate for a full-text analysis. Five of these concerned interventions that were not primarily Internet-delivered (eg, only text messaging). Two articles remained [23,24], but these were already included in the initial search.

### Data Extraction

Data were extracted on the target group, number of eligible articles, and the size of corresponding study samples. Our main interest was what the studies reported on the reach, effectiveness, and use of the interventions reviewed. Reported limitations and recommendations were extracted also. Some of the selected reviews partly contained studies that did not correspond with the objectives of this study (eg, non–Internet-delivered interventions). Only data reflecting the eligible interventions have been reported and these data are included in Multimedia Appendix 2.

### Quality Assessment

A quality assessment was performed using the AMSTAR tool as well as the more detailed and sensitive R-AMSTAR tool. These tools are especially designed to assess the quality of systematic reviews and meta-analyses. With the AMSTAR tool, such studies are assessed on 11 quality criteria (yes/no/can’t answer/not applicable); total scores can range from 0 to 11 [25]. The revised AMSTAR tool (R-AMSTAR) uses the same 11 criteria, but subdivides them into separate items, making the R-AMSTAR more sensitive. Total R-AMSTAR scores can range from 11 to 44 [26]. Both these tools have been validated [26-28].

## Results

### Characteristics of the Selected Studies

We included 41 papers [13,15,23,24,29-65] after article selection (Multimedia Appendix 2). Most studies were systematic reviews (27/41, 66%) and 16 reported meta-analyses (16/41, 39%) [13,15,30,33,35-41,43-45,52,59]. The studies were classified into 8 groups according to the behavior they targeted. The largest group targeted weight management, comprising 11 studies aimed at both dietary behaviors as well as physical activity [24,29,31,37,38,40,42,47-49,64]. All these were aimed at weight loss, and there were 5 reviews that also included interventions on weight maintenance [31,37,40,47,49]. Six studies included 3 or more behaviors [13,15,54,56-58]. The other groups included studies aimed at physical activity (6/41, 15%) [23,36,51,53,60,65], smoking (5/41, 12%) [30,35,39,45,46], alcohol use (5/41, 12%) [33,43,55,61,62], substance use including combined alcohol use and smoking (4/41, 10%) [34,44,52,63], and dietary behaviors (3/41, 7.3%) [32,50,59]. An additional manual search revealed a study on condom use (1/41, 2.4%) [41]. All studies were published between 2006 and 2012. Because we had broad inclusion criteria, study populations ranged from children aged 6 years [23] to people aged 50 years and older [29]. Most reviews were aimed at adults; 30 of 38 reviews (79%) reported age groups including adult populations. The mean of the AMSTAR score on overall quality of the included studies was 3.56 (SD 2.06). The mean of the R-AMSTAR score was 25.5 (SD 5.20). Item scores for all included studies are available in Multimedia Appendix 3.

### Reach

Most of the time, a very homogeneous sample of the population is reached. There is a strong female dominance, especially in weight loss/management interventions. Of the 18 reviews that reported gender distribution of the total sample, 16 reviews (89%) reported reaching more women than men [29-32,36,39,40,42,47,49,51,55,59,60,62,65]. Participants were predominantly highly educated [13], young, white [30,31], and living in high-income countries [32,33]. They were approached primarily through traditional offline recruitment efforts; however, some studies use online advertising or suggest to use more modern recruitment strategies [29].

### Effectiveness

Overall, Internet-delivered interventions seem to have the potential to achieve behavior change. However, effect sizes were small, rarely moderate [13,15,29,33-44,66]. Internet-delivered interventions compared to a no-treatment control condition had larger effect sizes than when compared with other interventions [13,30,45-47]. In some cases, often including face-to-face elements, the control was equally or more effective than the Internet-delivered intervention [24,31,37,42,46]. It is not exactly clear what effective elements were and for whom these were effective [15,40,46]. For some, indications of effectiveness were found. From this review, tailored feedback [13,24,33,43,47,48], use of theory [15,36,38], interactivity [30,38], goal setting [24], and combinations of online and in-person contact [32] emerge as noteworthy promising constructs. It remains to be explored what elements work in what situation, and in what combination [13,29,40,47-51]. Furthermore, it is also unclear when interventions become cost-effective [15,33,36,37,43-45,47,52,53]. Long-term effects are measured in a limited number of interventions [34,36,38,39,50-52]; these effects are often unknown and the results that are available show very limited sustained effects (≥6 months, following the RE-AIM framework individual maintenance standards). Studies indicate that effect sizes decrease with intervention length and postintervention or that behavior is not maintained at all because effect sizes were quite small initially [38,51,52]. Embedding an Internet-delivered intervention in existing structures (eg, schools, health care) might increase effectiveness [54,55]. Whether increased reach or use are the underlying driving forces behind this increased effectiveness is uncertain.

### Use

One of the largest problems in Internet-delivered interventions is low actual use. There is a wide variety of terminology used to describe use-related constructs (eg, adherence, exposure, and intervention attrition). Experimental research and theory development regarding intervention use is still in its infancy. Given the dose-response relationship between use and effectiveness, this is crucial to improve Internet-delivered interventions aimed at inducing behavior change. Factors suggested to stimulate the use of an intervention were sending reminders [40,54,56], incorporating professional support [54,56,57], and embedding interventions in existing structures [54,55]. Process evaluations should explore people’s user experience in order to be able to adjust interventions accordingly [58].

An illustrative description of the average Internet-delivered intervention states that a typical specimen is meant to be used once a week, is modular in setup, is updated once a week, lasts for 10 weeks, includes interaction with the system, a counselor, and peers on the Internet, includes some persuasive technology elements, and results in approximately 50% of the participants adhering to the intervention [56]. Lastly, an issue regarding the use of an intervention is that this is mostly not objectively measured, and if so, heterogeneously, very poorly or not at all described [56,57].

### Dietary Behaviors

The reviews on improving dietary behaviors primarily focused on younger populations (children, adolescents, young adults) [32,50]. One study performed a cost-effectiveness analysis and concluded that eHealth devices are unlikely to be cost-effective [59]. The studies showed small effects and the limited data on follow-up measures show that these effects were usually not maintained. The number of available reviews was small (3/41, 7.3%), and individual studies including follow-up measures were even scarcer (1/3, 33%). It was also recommended that the role of social support in Internet-delivered interventions shows promise and should be investigated more thoroughly [50].

**Table 1:** Quality assessment of included studies.

Reference	Behavior	Study design^a^	AMSTAR score^b^	R-AMSTAR score^c^
Harris et al (2011) [59]	Dietary behaviors	M	9	37
Hamel et al (2012) [50]	Dietary behaviors	SR	4	27
Lua et al (2012) [32]	Dietary behaviors	SR	1	18
Davies et al (2012) [36]	Physical activity	M	6	33
Lau et al (2011) [23]	Physical activity	SR	3	24
Laplante et al (2011) [65]	Physical activity	SR	4	26
Hamel et al (2011) [60]	Physical activity	SR	5	29
Van den Berg et al (2007) [53]	Physical activity	SR	2	24
Vandelanotte et al (2007) [51]	Physical activity	SR	1	23
Bewick et al (2008) [61]	Alcohol use	SR	3	25
Riper et al (2009) [43]	Alcohol use	M	5	31
Vernon (2010) [62]	Alcohol use	SR	1	18
White et al (2010) [55]	Alcohol use	SR	3	22
Riper et al (2011) [33]	Alcohol use	M	5	34
Myung et al (2009) [39]	Smoking	M	5	31
Shahab and McEwen (2009) [30]	Smoking	M	2	25
Civjlak et al (2010) [45]	Smoking	M	6	33
Hutton et al (2011) [46]	Smoking	SR	3	27
Chen et al (2012) [35]	Smoking	M	10	38
Noar et al (2009) [41]	Condom use	M	4	28
Weinstein et al (2006) [31]	Weight management	SR	1	18
Saperstein et al (2007) [48]	Weight management	SR	1	17
Maon et al (2012) [38]	Weight management	M	4	24
Fry et al (2009) [49]	Weight management	SR	2	20
An et al (2009) [64]	Weight management	SR	3	21
Neve et al (2010) [40]	Weight management	M	6	31
Manzoni et al (2011) [47]	Weight management	SR	2	18
Aalbers et al (2011) [29]	Weight management	SR	2	25
Kodama et al (2012) [37]	Weight management	M	5	32
Coons et al (2012) [24]	Weight management	SR	2	23
Norman et al (2007) [42]	Weight management	SR	2	22
Champion et al (2012) [34]	Substance use	SR	4	24
Lehto et al (2011) [63]	Substance use	SR	4	26
Rooke et al (2010) [44]	Substance use	M	2	23
Tait et al (2010) [52]	Substance use	M	3	23
Kelders et al (2012) [56]	Multiple behaviors	SR	3	24
Donkin et al (2011) [58]	Multiple behaviors	SR	1	21
Brouwer et al (2011) [57]	Multiple behaviors	SR	2	22
Crutzen et al (2011) [54]	Multiple behaviors	SR	4	27
Cugelman et al (2011) [13]	Multiple behaviors	M	5	27
Webb et al (2010) [15]	Multiple behaviors	M	3	23

^a^M: meta-analysis; SR: systematic review.

^b^AMSTAR total score range 0-11.

^c^R-AMSTAR total score range 11-44.

### Physical Activity

Most reviews on physical activity conclude that when studies are effective, these show modest effect sizes with decreasing effectiveness during follow-up [36,51,53,60]. A large meta-analysis showed that longer intervention duration, the use of social cognitive theory, the use of educational components, and regularly updating the content of the intervention had significant effects on physical activity levels [36]. There was a reoccurring call for research on increasing intervention use [23,36,51].

### Alcohol Use

In alcohol abuse prevention, the risk profile of users is thought to be related to the effectiveness of the intervention [61]. It is suggested that brief interventions may be more effective for high-risk participants than low-risk participants. Several studies focused on the use and content of feedback, in which findings on normative and personalized feedback seemed promising, but still inconclusive [43,62]. It was suggested to add a face-to-face component as an adjunct to Internet-delivered interventions to increase effectiveness, but effectiveness is not yet confirmed by research [33]. Use is a problem [43,61]; 1 review showed that women, people married or living with a partner, and those without children were more likely to complete the program [62]. Collecting user feedback to tailor interventions could increase this outcome [62].

### Smoking

Almost all reviews aimed at smoking cessation recommended research that focuses on the use of the intervention, which is suggested to be improved by assessing barriers to participation [46]. Effectiveness may be improved by involvement of users in the design of interventions [30,35]. Adolescents form a target group in need of extra attention [35,39,46]. Biochemical measures to measure smoking behavior may be more accurate, but effectiveness appears to remain the same regardless of the method [30,39,46]. Increasing interactivity and message tailoring seemed effective strategies [30,35,46]. In smoking cessation, motivated participants, which were often more females than males, tended to show larger effects on behavior change [30].

### Condom Use

There was only 1 systematic review available on condom use, which discussed 3 Internet-delivered interventions [41]. Individualized tailoring and the use of the stages of change model were related to effectiveness, but these were overall conclusions, not specified for Internet-delivered interventions.

### Weight Management

In the weight management reviews, the majority of studies reported a strong overrepresentation of women (7/11, 64%), which makes a generalization of the effects to men questionable [31,47,49]. Evidence points toward the effectiveness of including a face-to-face component in Internet-delivered weight loss interventions. When personal contact is part of the intervention, results generally showed that effect sizes and use were higher [31,40,47,48]. When personal contact was not part of the intervention, reverse effects were seen [37]. There was, however, no conclusive evidence. Interactive elements (eg, online peer support or forums) were shown to increase effectiveness; however, use was very limited [29]. Some studies did not find a difference between Internet-delivered and traditional (face-to-face) interventions [24,31,38,40,47]. It was suggested that a difference was hard to find because of the high attrition rates [24] or that this finding might reflect an equal effectiveness, which could mean opportunities to increase interventions’ cost-effectiveness [37]. Five reviews (5/11, 45%) included weight maintenance interventions in which the weight maintenance studies were always outnumbered by the weight loss studies [31,37,40,47,49]. The limited findings indicate face-to-face interventions are more effective than Internet-delivered interventions.

### Substance Use

Most studies in the reviews on substance use were aimed at adolescents or young adults, especially with regard to cigarette smoking behavior [34,52,63]. It is not clear what elements of the interventions were effective, but suggestions were made toward parental involvement, number of sessions, so-called booster sessions, normative education, resistance skills training, and reducing positive expectancies [34]. Also within these behavior domains there was a demand for research that compares Internet-delivered with in-person interventions or combinations of the 2 [44,52]. One meta-analysis concluded that Internet-delivered interventions were significantly less effective than offline computer-delivered interventions [44].

### Multiple Behaviors

As opposed to the other review sections, effectiveness was not always the main outcome when multiple behaviors were considered. Reviews also focused on intervention use, which has been shown to be related to effectiveness. For instance, more adherent participants of weight loss programs lost more weight [58]. Because effectiveness was the most commonly reported outcome, some reviews experienced difficulties collecting their data due to poor reporting on other constructs than effectiveness [54,56,57]. Several reviews showed that nonadherence was a major problem of Internet-delivered interventions in particular, and a large barrier to effective interventions [58]. Frequency of interaction (eg, email and/or phone contact with visitors, but also reminders), dialog support, updates [56], targeted/tailored communication, monitoring of behavior change, professional and peer support, interactive and easy accessible content, conditional progress, incentives, and embedment in a social context all seemed to increase use [54].

With regard to effectiveness, the reviews found that primary task support [56], extensive use of theory, inclusion of more behavior change techniques, elaborate interaction with participants, and training in stress management and general communication skills showed significant results on behavior change [15]. Intervention duration was negatively correlated with impact [13]. There was low use of interactive elements with peers and with professionals [54].

## Discussion

### Principal Findings

The sizable growth of Internet-delivered interventions can be recognized when comparing the 41 reviews found in this study to the 9 individual studies found in a study with similar objectives conducted in 2005 [22]. Almost half of the reviews on Internet-delivered interventions (20/41, 49%) were aimed at overweight-related behaviors (physical activity and dietary behaviors), reflecting a research focus congruent with health priorities [67]. For smoking, the leading cause of preventable deaths in the United States [68], 9 reviews were found. For condom use, data on Internet-delivered interventions were very limited; only 1 review was available [41] focusing on computer-delivered interventions in general (ie, not necessarily Internet-delivered).

This review confirms the previously mentioned statement that within the public health field, there is a strong focus on effectiveness. It is reported most often and thoroughly, but effect sizes appear to be small, variable, and behavior change appears to be unsustainable at follow-up measures. Effect sizes may be small, but it should be noted that the Internet is a delivery mode with an unrivaled potential reach and this may still enhance public health impact [69]. Estimates of actual reach for Internet interventions are lacking [17]. The intended reach of Internet-delivered interventions is varied, aiming at a diverse population with respect to gender, socioeconomic status, and ethnic background, but the actual reach is undiversified; mostly the female, Caucasian, highly educated part of the population is reached, corresponding to previous findings [70]. It is also desirable to reach high-risk participants; however, these are not necessarily attracted at the same rate as low-risk participants [71]. Another limitation of the available studies is that most research is predominantly aimed at high-income populations [32,33], which makes generalization to low- and middle-income countries questionable. Although the potential reach of Internet-delivered interventions is virtually infinite, this review shows that, at least in terms of reach, there is still much to be gained.

Effect sizes are small, variable, and not sustainable. But what determines effectiveness? It is often not clear what the relevant active ingredients are for effectiveness [72,73], and there is a clear call for elucidation on this subject from this review. The current data show indicative evidence for the effectiveness of tailored feedback, use of theory, interactivity, goal setting, and combinations of online and in-person contact. For several behavior change techniques, there seems to be some evidence, not all equally conclusive. Some well-substantiated evidence can be found for the effectiveness of message tailoring [74,75], but variations between interventions exist in the operationalization, and effectiveness is not guaranteed in all cases [76]. A large meta-analysis found that extensive use of theory and the use of multiple behavior change techniques predict effectiveness in Internet-delivered interventions [15]. Including face-to-face elements is recommended or found to increase the use or effectiveness in weight loss interventions [31,37,40], alcohol use interventions [33], and smoking cessation interventions [35]. This could, however, have negative implications for reach. Including face-to-face support undermines the potentially high reach of Internet-delivered interventions, because of a high therapist burden. It is recommended to find a balance between face-to-face elements and self-guided Internet-delivered material [30,31,33,35,37], also in the light of cost-effectiveness [37]. Adjunctive designs have been applied to Internet-delivered interventions; however, it is not yet discerned what elements cause the effect of an intervention and whether these adjunctive elements contribute and to what extent [77]. Some reviews indicated that interventions show improved effectiveness when they are embedded in an existing structure, such as schools or health care. This may not only improve effectiveness, but may improve reach and use as well. It is also suggested to use Internet-delivered interventions as a part of a stepped-care model [78]. In this way, Internet-delivered interventions could serve as a first step in which individual needs are assessed with respect to the necessary amount of support, time, and expertise [79].

Internet-delivered interventions are likely to be less costly than a face-to-face intervention and this is an oft-cited reason for delivering an intervention online [11]. This reasoning probably holds for fully automated systems, but a fair point is made when considering that Internet-delivered interventions can still contain a substantial amount of human involvement [79], which makes assumptions on cost-effectiveness less certain. Research on cost-effectiveness was recommended repeatedly in the reviews considered in the current study. A study answering this call concluded that cost-effectiveness is hard to determine, especially due to a lack of data [80].

Data on the interventions’ use were poorly reported in most studies; in some cases, these data were completely lacking. These are missed opportunities, because Internet-delivered interventions in particular have the technological advantage to be able to provide more insight into intervention use [58]. From the currently reported data, it is shown that there is particularly low use. The phenomenon defined as the discontinuation of eHealth application use, called *the law of attrition*, is considered to be “one of the fundamental characteristics and methodological challenges in the evaluation of eHealth applications” [12]. In our own findings, the higher attrition rates in Internet-delivered compared to traditional interventions is most clearly illustrated by the review on condom use, in which the 2 trials with the poorest use were delivered online [41]. Participants’ nonusage of an intervention can be explained from a reversed diffusion of innovations model [12,81]. The diffusion of innovations model is explained by a symmetric curve depicting the proportion of a population adopting an innovation and their motivations; the reversed model would reflect on the discontinuation of using an innovation (in this case, an intervention). Following this line of reasoning, factors influencing adoption may be used in Internet-delivered interventions to prevent low usage. There are a wide range of factors thought to stimulate intervention use. Christensen and Mackinnon [82] point out the importance of user characteristics and preferences. A review of adherence in 3 Internet-delivered trials showed a positive association for higher self-efficacy, having less smoking friends, older age, being female, and a higher education with an increased use of the intervention [83], but also interaction with a counselor, more frequent intended usage, more frequent updates, and more extensive employment of dialog support were found to be predictors of improved use [56]. Engaging users is also thought to be improved by the transition from a more static, structured, developer-defined intervention content to less structured, more user-defined interactions [84,85]. Research on the use of Internet-delivered interventions is a relatively young and sometimes overshadowed endeavor; therefore, most findings are not yet confirmed by a substantive body of evidence. It is important that research is contributing to this debate [82], because decreased intervention use has been shown to negatively affect health behavior change [58] and the findings of the current study further underline the relevance of this problem.

### Recommendations for Future Research

This review shows that a substantial amount of research has been done, but we found some reoccurring research needs, which will be discussed in terms of reach, effectiveness, and use.

The interventions’ reach is found to be undiversified and it is hard to reach high-risk groups. Participant recruitment can be done offline as well as online. Although reach seems to be much larger online, online recruitment can be a disappointing venture, even when targeting a young audience [86]. Recruitment strategies should be aligned with users’ search strategies, which have to be studied first. Another strategy to increase reach is to create interest for the intervention. A lack of interest for Internet-delivered lifestyle interventions is identified by Lieberman and Massey [87], who developed a motivational Internet application that was used to increase treatment interest in individuals with drinking problems not receiving treatment. Including a meta-intervention, which can be described as a small intervention prior to the actual intervention, there was increased participation of high-risk participants in an HIV-prevention intervention [88]. It is also found that motivation for behavior change and curiosity regarding the content of the intervention appear to be important for a first visit to an Internet-delivered intervention [89]. An extension of this line of research is warranted.

The largest gap in research on effectiveness seems to be that it is not known what intervention elements are effective and under what conditions. Future research should entail experimental studies focusing on testing isolated ingredients of Internet-delivered interventions. Effect evaluations on complete interventions are widely available and interventions proven to be effective could provide a starting point to disentangle effective ingredients. These may also be found outside the scope of Internet-based strategies, in adjunctive designs. Here, online and offline strategies are combined and it would be very interesting to investigate optimal combinations, possibly also in the context of cost-effectiveness. Interventions should include user profiles with information on a wide range of user characteristics. Such data are not only suitable for developing tailored advice, but could also shed light on effectiveness for subgroups of the population (eg, high-risk groups) to answer the question: what is effective for whom?

Discontinued or suboptimal use of the intervention is a widely recognized problem, also evident from the current review. There is a strong need for strategies that can be employed to increase use. User involvement in the creation of the intervention may be one of these strategies to keep users engaged. Some work has been done on this topic [89], but these findings need replication and further study. Moreover, there is a need for theory-driven experimental studies focusing on use of Internet-delivered interventions [90].

For all areas of research, reviews recommend better data reporting because inadequate data reporting posits unnecessary limitations to research. This is especially the case when conducting a meta-analysis or review, or in the case of data on the reach or use of interventions. Full disclosure could be a solution, because it stimulates scrutiny and replication of research findings, and improves meta-analytical research [91,92]. Moreover, the current technological developments should be employed more exhaustively. Therefore, cooperation with other disciplines is warranted to be able to develop modern, well-designed interventions. This intensified use of technology could not only be applied to data sharing, but also to increase effectiveness of interventions and to provide more insight into reach and use. Regarding the latter, there are some unused opportunities in terms of data collection [58]. The Internet offers possibilities to collect a wide range of valuable data regarding intervention use (eg, log-ins, page views), which is readily available and should be disclosed.

### Limitations

Because of the substantial amount of data, we decided to perform a review of reviews. There are some weaknesses inherent to secondary analyses. It is inevitable that data and details are lost in order to obtain a more robust overview. The quality of our data is directly dependent on the reporting and execution quality of the reviewed studies. We performed a quality assessment on the included studies. The scores approached the lower end of the scales, but were comparable to those found in previous studies [93]. Although the search was executed with the greatest consideration, it is possible that some publications or data may have been missed. This might be because only the primary search engine for this type of research was used to collect our data (ie, PubMed), although we also checked reference lists for completeness of our review, or because publications in English were solely considered in our review. Although we tried to be as elaborate as possible in the methodology, it should be acknowledged that this study may be affected by subjectivity bias because of the nature of the study design (ie, a systematic review of reviews) and quantitative data being available limitedly. Another possible bias lies in the fact that we did not correct for studies appearing in several of the included reviews. The result may be that some of the same evidence is covered more than once. Because we did not perform quantitative analyses, the resulting bias is estimated to be limited.

Despite these possible biases, we believe that the systematically collected data contributes to our understanding and to a general overview of what research has been done and what research still needs to be done.

### Conclusion

This review provides an overview concerning research on online prevention aimed at lifestyle behaviors. The findings of this research show that reviews are effect-oriented, but interventions show small, unsustainable effects on behavior change. Research on reach and use of interventions is less advanced and needs more scientific input.

## Multimedia Appendix 1

Search query.

## Multimedia Appendix 2

Description of included studies.

## Multimedia Appendix 3

Quality assessment of included studies.
