# Supplementary material for: Online Prevention Aimed at Lifestyle Behaviors: A Systematic Review of Reviews
Source: J Med Internet Res. 2013 Jul 16;15(7):e146. doi: 10.2196/jmir.2665 (PMC3714003; doi:10.2196/jmir.2665)
Supplement: Supplementary file 1 [file jmir_v15i7e146_app1.pdf]

Multimedia Appendix 1. Search query

(e-health(Title/Abstract) OR ehealth(Title/Abstract) OR internet*(Title/Abstract) OR web*(Title/Abstract) OR online*(Title/Abstract)) AND ("physical activity"(Title/Abstract) OR exercis*(Title/Abstract) OR sport(Title/Abstract) OR exertion(Title/Abstract) OR training(Title/Abstract) OR "energy balance"(Title/Abstract) OR smok*(Title/Abstract) OR tobacco(Title/Abstract) OR cigarette(Title/Abstract) OR alcohol(Title/Abstract) OR drinking(Title/Abstract) OR AOD(Title/Abstract) OR substance(Title/Abstract) OR nutrition(Title/Abstract) OR food(Title/Abstract) OR eat*(Title/Abstract) OR weight(Title/Abstract) OR obesity(Title/Abstract) OR overweight(Title/Abstract) OR diet(Title/Abstract) OR dieta*(Title/Abstract) OR adiposity(Title/Abstract) OR sexua*(Title/Abstract) OR "safe sex"(Title/Abstract) OR condom(Title/Abstract) OR HIV(Title/Abstract) OR aids(Title/Abstract) OR STI(Title/Abstract) OR STD(Title/Abstract) OR STIs(Title/Abstract) OR STDs(Title/Abstract) OR behav*(Title/Abstract) OR health(Title/Abstract) OR lifestyle(Title/Abstract) OR preven*(Title/Abstract) OR intervention(Title/Abstract)).
