# Supplementary material for: Online Prevention Aimed at Lifestyle Behaviors: A Systematic Review of Reviews
Source: J Med Internet Res. 2013 Jul 16;15(7):e146. doi: 10.2196/jmir.2665 (PMC3714003; doi:10.2196/jmir.2665)
Supplement: Supplementary file 2 [file jmir_v15i7e146_app2.pdf]

Multimedia Appendix 2. Description of included studies

Study characteristics	*RE-AIM*	*Limitations*	*Recommendations*
DIETARY BEHAVIORS			
Harris et al. (2011)<br>Study type: M[1]<br>Articles[2]: 22/43<br>Total sample size[3]: 14230/ 21872<br>Target group: Adolescents or adults	[R] Of the studies targeting adults, the majority of studies had participants with a mean age of 40-49 years (n=43). Most studies contained more women than men.<br>[E] Nine studies examined fruit and vegetable intake, with WMDs available for 6 of these studies. WMDs were (0.14 CI(-0.05-0.33); 0.21 CI(-0.09-0.51); 0.13 CI(-0.42-0.68); 0.33 CI(0.09-0.57); 1.50 CI(-0.06-3.06)). Five studies looked at fat intake, with WMDs available for 3 studies (0.55 CI(-5.59-6.79); 1.84 CI(-4.47-7.95); 6.80 CI(-19.66-33.26)). One of these studies (last WMD) also looked at saturated fat intake (3.90 CI(-2.86-10.66)). Two studies reported on fiber intake, only one WMD was available (0.57 CI(-1.01-2.15)). Energy intake was addressed in three studies of which 2 WMDs were available (4.40 CI(-233.71-242.51); 377.00 CI(-189.19-943.19)).<br>A cost-effectiveness analysis broadly suggests that the e-learning devices are unlikely to be cost-effective at conventional levels of ￡20,000–30,000 per QALY gained.	There was unexplained heterogeneity in trial results; the exploration of potential sources of heterogeneity was not exhaustive, owing to a limited number of studies in the review (and hence power).	Further clinical trials of individual e-learning interventions should not be undertaken until theoretically informed work, which addresses the question of which characteristics of the target population, target behavior, content and delivery of the intervention are likely to lead to positive results, is completed. This work would include:<br>- reviews of available behavior change theoretical frameworks and the empirical data to support each approach<br>- research in behavior change techniques (linking theory to techniques) to provide empirical data to help understand which techniques are effective, and under which conditions<br>- cohort and other study designs which actively map and explore the pathways of change in outcomes among users of the intervention.
Hamel et al. (2012)<br>Study type: SR[4]<br>Articles: 10/15<br>Total sample size: NR[5]<br>Target group: Children & adolescents	[R] Of the 10 web-based interventions, six were school-based.<br>[E] Six of the ten web-based interventions resulted in significant improvements in eating behavior, and/or diet-related physical outcomes. The studies showed lowering BMIs, reducing % dietary fat, improved juice, fruit and vegetable consumption, and less body fat. The significant effects on dietary behavior were not maintained in the studies that included post-intervention follow-up.<br>[AIM] 7 of 11 school-based, computer- *and* web-based interventions showed significant results, this might be an indication of the ease with which web- and computer-based interventions can be implemented in schools instead of traditional print nutrition education.	All interventions relied on self-reported measures, either alone or in addition to a diet-related physical measure. Only three studies employed follow-up measures.	Future research involving computer- and web-based interventions needs to examine the influence of social support from teachers and classmates in an educational setting, and also from family, friends, and other media sources on the improvement and maintenance of eating behaviors and diet-related physical outcomes. Future investigations need to examine the various nuances that can potentially influence the effect of tailoring in computer- and web-based interventions.<br>Post intervention measures should be at least 6-12 months. Maintenance strategies must be implemented to sustain the positive behavior change. School-based interventions and intervention with tailored or personalized feedback should be included in the academic curriculum for children and adolescents.<br>Web-based assessments have been recommended to compliment and support physicians and nurses in their efforts to promote health. On the basis of this review, the following recommendations for future research involving computer- and web-based interventions include:<br>(1) integrate interventions in a school setting;<br>(2) incorporate individually tailored or personalized feedback;<br>(3) use physical measures whenever possible to obtain an objective assessment; and

Study characteristics	*RE-AIM*	*Limitations*	*Recommendations*
			(4) incorporate post-intervention follow-up efforts to maintain any change. Future reviews of these types of interventions should continue to evaluate the influence of particular intervention elements on the outcomes.
Lua et al. (2012)<br>Study type: SR<br>Articles: 4/14<br>Total sample size: 783/ 1668<br>Target group: 18-25 years	[R] Participants were college or university students in developed countries. More female students than males.<br>[E] Of the four studies, one showed that a combined feedback and online intervention resulted in lowered BMI. One study showed increased self-regulation and self-efficacy in consuming 3 servings of dairy per day and the last study, another showed effectiveness of nutrition education on promoting changes in health behaviors. The last improved body satisfaction, but showed no results for eating disorders.	There were 2 times more males than females in the overall study sample size. Females may have a stronger motivation than males. The common usage of cross-sectional design has its drawbacks. Some studies had small sample sizes. Reliability and validity of assessment tools were not comprehensively reported. Number of available studies was small. Studies from developing countries were lacking.	Undesirable dietary norms should be addressed at earlier ages during university years preferably through individuals' routine learning environments. Nutrition education is a well-suited technique to improve both students' dietary habits and their awareness of overall health.<br>Research should focus on the development of nutrition education tools, which are not only effective but also interesting and practical for the current generation of students.<br>Future studies should also enroll larger samples, with the provision of sample size calculations, and a more balanced gender representation. It is suggested that further trials of similar nature, with improved methodology and in less-developed countries, are highly important.
**PHYSICAL ACTIVITY**			
Davies et al. (2012)<br>Study type: M<br>Articles: 34<br>Total sample size: 9,638<br>Target group: Adult population	[R] 65% of the studies represented a general/ overweight population, the other studies were aimed at a not-healthy population. 26% of the studies had participants classified as inactive, the other studies did not report on physical activity level. The mean age was 43.06, 65% was female and 92% Caucasian.<br>Health status<br>General population [*d*=0.11 CI(0.06-0.17)], Overweight [*d*=0.28 CI(0.07-0.48)]).<br>Physical activity level<br>Not screened for [*d*=0.12 CI(0.08-0.16)], Sedentary [*d*=0.37 CI(0.21-0.52)].<br>The samples were largely Caucasian and well-educated. 50% of population had some sort of condition (overweight, type 2 diabetes, metabolic syndrome, etc.).<br>[E] Overall effect [*d* = 0.14 CI(0.09-0.19)]<br>Intervention duration<br>0-6 weeks [*d*=0.11 CI(0.03-0.19)], 7-12 weeks [*d*=0.13 CI(0.08-0.19), 13+ weeks [*d*=0.21 CI(0.09-0.33)]<br>Use of Social Cognitive Theory<br>- Yes [*d*=0.20 CI(0.14-0.27)]<br>- No [*d*=0.09 CI(0.03-0.15)]<br>Use of Educational Components	The study did not evaluate the impact of intervention features on program engagement. Self-report measures were used, which are not objective. The sample was largely Caucasian and well-educated and could be more representative.	Small studies even with non-significant results should also be published to avoid publication bias. Future research should include cost-effectiveness studies, long-term follow-up measures, be high quality (RCT), have a diverse sample and focus on increasing engagement and retention.

Study characteristics	*RE-AIM*	*Limitations*	*Recommendations*
	- Yes [*d*=0.20 CI(0.14-0.26)]<br>- No [*d*=0.08 CI(0.01-0.14)]<br>Updated content<br>- Yes [*d*=0.19 CI(0.13-0.26)]<br>- No [*d*=0.10 CI(0.04-0.16)]<br>Studies with a larger sample size had a smaller effect [*d*=0.12 CI(0.07-0.16)]. The overall mean effect for sustained physical activity at least 6 months post-intervention (n=11) resulted in a small but significant effect size (d = 0.11, p < 0.01)		
Lau et al. (2011)<br>Study type: SR<br>Articles: 4/9<br>Total sample size: 987/1456<br>Target group: Children and adolescents, aged 6-18.	[R] 48.1% was female.<br>[E] Only 1 out of 4 interventions had a significant effect, only in males (d = 0.95).	Only one study employed objective measures and conducted long-term follow-up. Intervention exposure rate was improperly reported and measured.	Future studies need to adopt objective and valid instruments to measure intervention exposure rates. It is recommended to intervene for a longer duration.
Laplante et al. (2011)<br>Study type: SR<br>Articles: 20/31<br>Total sample size: 7435/ 9877<br>Target group: Adults and children (7-60s)	[R] 7 studies only female, 1 only male. Very heterogeneous interventions in terms of target audience. Mean age ranged from 10-52.<br>[E] 14 studies showed significant effects on PA, however 5 of these found no difference between intervention and control group. Six studies found no effect at all.	Almost all studies had a Caucasian sample. Outcomes reported in the paper refer to a follow-up survey conducted some time after the intervention was completed.	Future studies could include more racial diversity and increase the use of objective measures. Also a more systematic and rigorous approach is needed (power analyses, theory to guide interventions, use of pure control groups instead of comparison groups).
Hamel et al. (2011)<br>Study type: SR<br>Articles: 7/14	[R] 5 studies were all-girls, one all-boys and 2 were mixed. 4 studies were racially diverse, two studies were 100% African-American, 2 studies did not report race.<br>[E] 3 studies showed a significant effect on PA, 2 showed an increase, but no difference with the control group. Two	Limited number of studies testing effectiveness, self-reporting of PA is less reliable, half of the studies reported were not RCTs, and there is a lack of interventions for boys.	Home-based studies indicate that involving parents is effective. More research should be done on individual tailoring and counseling. The use of theory is important. Post-intervention efforts are needed to sustain increased in PA. There is a need for greater intervention intensity.

Study characteristics		*RE-AIM*	*Limitations*	*Recommendations*
Total sample size	1390/ 6236	found no effects for internet interventions at all.		
Target group	(Pre)adolescents (8-18 years of age)			
Van den Berg et al. (2007)		[R] One study was aimed at sedentary adults. Mean age varied from 42.8-48.5 years. Mean percentage of males ranged from 11-59%.<br>[E] 2 studies found a significant effect for the intervention group. Three studies found no significant between group differences.	Number of eligible studies was limited; many studies used indirect measures (e.g. weight change, heart rate etc.). Baseline physical activity levels were different or not measures. The contents of the control intervention differed widely.	Future research should evaluate the minimal duration of PA interventions in order to produce long-term PA behavior change and also examine only one intervention component at once (e.g. personalized supervision). Cost-effectiveness studies should be done to establish the exact surplus value of Internet delivery.
Study type	SR			
Articles	5/1082			
Total sample size	3501/39			
Target group	Adults (18+)			
Vandelanotte et al. (2007)		[R] 66% of all participants was female, 84% was highly educated.<br>[E] Modest evidence for efficacy of web-delivered PA interventions. 8 of 11 studies showed positive effects on PA of which three did not differ from control group. Three studies found no effect. Mean effect size (calculated for 8 studies of which 2 are not included in this analysis) was 0.44.<br>Interventions with more than 5 communications were more successful, with 7 of 9 reporting positive outcomes. There was a clear decrease in efficacy when time to follow-up increased. Six studies measured follow-up, of which 4 found a positive effect. When physical activity was measured before the end of the intervention, six of seven studies showed positive results (86%). When it was measured immediately after the end of the intervention, five of twelve studies showed positive outcomes (42%). Five studies measured physical activity following the end of the intervention: one within 3 months (negative outcome), three between 3 and 6 months (two with positive outcome), and one longer than 6 months (positive outcome).<br>[AIM] Attrition rates ranged from 9-69%, with an average	Only one third of the studies reported objective data on website usage. It was not possible to compare specific intervention elements. The results are less well generalizable to less-educated samples. Not using Intention-to-treat might introduce bias into the results. The power of some studies was suboptimal. Women were overrepresented in the samples. There could be a publication bias present in the results. It is possible that only those participants that were very motivated to increase their physical activity were willing to keep using the website-delivered intervention and therefore had better outcomes.	Methods to increase website use and exposure to intervention materials and subsequently increase engagement and retention of participants need to be developed. Gathering objective data on website usage is technically possible and is an important indicator of intervention effectiveness that should be used in all future trials. Increasing interactivity in interventions alone will not be enough, because the current review found low ES in interactive interventions. Computer tailoring is a potential strategy to increase efficacy of website-delivered interventions that need to be further explored. Intervention elements should be more directly comparable. The optimal duration and intensity of interventions should be explored. To capture true maintenance, PA should be measured at least 6 months after the end of the intervention. Data with and without Intention-to-treat should be presented. Sample sizes should larger en samples should be more representable.
Study type	SR			
Articles	11/15			
Total sample size	4355/ 4845			
Target group	Adults			

Study characteristics	*RE-AIM*	*Limitations*	*Recommendations*
	of 27%. Studies reporting in web site usage report low exposure, with decline as intervention progressed.		
**ALCOHOL USE**			
Bewick et al. (2008)<br>Study type: SR<br>Articles: 9/10<br>Total sample size: 4665/ 8540<br>Target group: NR	[R] Mean age ranged from 19.9-40.9 year; percentage female ranged from 44-77%.<br>[E] It is suggested that Electronic Screening and Brief Interventions may be more effective for high risk-participants than low risk participants. Research designs are described to be weak; the only strong design did not find significant effects. Most studies reported data in favor of the comparison group. Results on personalized feedback appeared to be more effective than information only results in one study, another study was inconclusive toward this respect.	Within the reviewed studies, there was a lack of rigor within the research designs employed.<br>1) outcome measures used were heterogeneous,<br>2) relatively small sample size at follow-up,<br>3) large standard deviations with the data appearing to be skewed and log transformed scores were only available in one instance,<br>4) lack of controls within the majority of the studies identified, and<br>5) in some cases, there were large confidence intervals around the effect size. All of the identified effectiveness studies focused on web-based interventions targeted at the North American or New Zealand student population.	There is a need for further research to understand the relationship between levels of alcohol consumption and effectiveness of any intervention administered. Given the potential ability for web-based interventions to reach a wide audience at low costs, it would appear prudent to ensure that the efficacy of such interventions is evaluated before making them available to the public via the world wide web. Future research should examine the generalizability of the results to other settings and populations.<br>Further research is needed to gain detailed insight into what elements of personalized feedback are effective and whether interventions are likely to be effective for both high and low-risk drinkers.
Riper et al. (2009)<br>Study type: M<br>Articles: 3/14<br>Total sample size: 847/3682<br>Target group: Young and adult problem drinkers	[E] The effect sizes for the web-delivered interventions were [*d*=0.262 CI(-0.102-0.626; *d*=0.293 CI(0.107-0.478); *d*=0.230 CI(-0.044-0.504)]. Personalized normative feedback was as effective as multi-component personalized feedback.<br>[AIM] Loss to follow up ranged from 11-32%.	Some studies had drop-out rates above 30%. All studies relied on self-reported alcohol-consumption measures, their validity improves in an online setting however.	Further research is required to investigate the (long-term) effectiveness of normative personalized feedback and personalized feedback in the context of a stepped-care approach. Also cost-effectiveness and implementation in other settings (e.g. primary care) should be researched.
Vernon (2010)<br>Study type: SR<br>Articles: 6/8<br>Total sample size: 12036/ 12137<br>Target group: General public	[R] 3 interventions were conducted through the workplace (>70% female), remainder targeted at general public (50% female). Users of online services with greater problems are the ones who utilize these services most thoroughly.<br>[E] All but one intervention showed a significant improvement on at least one of the drinking outcomes studied. ES ranged from 0.40-1.19.<br>[AIM] Women, people married or living with a partner, and those without children are more likely to complete the program. Drop-outs were at greater risk for alcohol dependency and harm than completers.	Cost-effectiveness was impossible to comment on due to a lack of reported data. None of the reviewed studies controlled for additional forms of help sought by participants. It is unclear how well the results can be generalized, because the number of studies reviewed was relatively small and user characteristics vary considerably.	It is shown that more people participate in an online intervention when this is coupled with an assessment. Researchers could study the most common phrases used to find online alcohol services, so that these services are easier found. Feedback using norms from clinical populations are not appropriate in addressing the general public. Internet services can reach greater numbers when translated into minority languages. Measurements should be done on user feedback. This could be used to tailor future interventions. Current research would benefit from greater standardization and the use of well-validated alcohol measures (e.g. AUDIT, DrInc, SCORATES).

Study characteristics	*RE-AIM*	*Limitations*	*Recommendations*
White et al. (2010)<br>Study type: SR<br>Articles: 17<br>Total sample size: 8864 (2 studies not reported)<br>Target group: NR	[R] 12/17 studies reported interventions aimed at a student population. 11/17 studies targeted at-risk, heavy or binge drinkers. Mean age of all studies ranged from 18.1-48.2 and percentage females ranged from 27.6-77.9% (mean 54.5, median 52%).<br>[E] Based on 5 RCTs ES for alcohol units ranged from 0.02-0.81 (median 0.54). Overall effect (7/17 studies) ranged from -0.26-0.88. Online alcohol interventions bring out small but meaningful differential reductions in 10-gram alcohol unit consumed, blood alcohol concentration, and a range of other outcome measures.<br>[AIM] Retention rates ranged from 38.9-100% in intervention groups. Median retention was 83.4 at 1 month, 74.5 at 3 months and 74.5 at 6 months.	The majority on studies is on student samples and these may not be representative for the general population. Generalization is also hampered by the brevity of the intervention description in published papers, variable intervention uptake, completion rates, heterogeneity of outcome measures and follow-up periods. The current research evidence is fragmented and requires greater methodological rigor.	More research is required on the clinical appropriateness and usability of online health technologies, especially in non-student contexts. Future research should incorporate economic analyses. Researchers should respond rapidly to new technological advances, but during transfer of interventions to new delivery modes key effective ingredients of the intervention should be preserved. Engagement is a problem; internet interventions could be interfaced with targeted marketing campaigns or embedded in routine primary care. Future research should focus on most effective marketing and widespread dissemination of interventions.
Riper et al. (2011)<br>Study type: M<br>Articles: 7/9<br>Total sample size: 1452/1553<br>Target group: Adult problem drinkers (18+) excluding student populations	[R] All studies were conducted in high-income countries.<br>[E] <u>All studies</u><br>- All studies g=0.44 CI(0.29-0.50)<br>- Outliers excluded g=0.39 CI(0.23-0.57)<br><u>Type of treatment</u> (differed significantly)<br>- E-personalized normative feedback g=0.27 CI(0.11-0.43)<br>- E-self-help g=0.61 CI(0.33-0.90)<br><u>Type of analysis</u><br>- Intention-to-treat g=0.37 CI(0.21-0.54)<br>- Completers-only g=0.48 CI(0.11-0.86)<br><u>Type of venue</u><br>- Home-setting g=0.47 CI(0.25-0.69)<br>- Other setting (research, health center, workplace) g=0.39 CI(0.15-0.63)<br><u>Sample size</u><br>- Small g=0.36 CI(0.19-0.52)<br>- Large sample size g=0.52 CI(0.14-0.91)<br><u>Type of control condition</u><br>- Alcohol leaflet g=0.35 CI(0.21-0.48)<br>- Assessment only g=0.12 CI(-0.84-1.07)<br>- Waitlist control g=0.77 CI(0.19-1.34)	Results can only be generalized to self-referred adult problem drinkers in high-income countries recruited via the media (high readiness to change). High attrition may have biased the results.	Further research is recommended to explore evaluation and implementation of e-self-help interventions to low- and middle income countries. There is a need for more evaluations of the clinical outcomes and the cost-effectiveness of online screening instruments and interventions. Future studies should shed light on whether e-self-help interventions produce similar or better results when extended with face-to-face components and on whether they could serve as alternatives or adjuncts to face-to-face treatments in primary care settings.
**SMOKING**			
Myung et al. (2009)<br>Study type: M<br>Articles: 8/22	[R] Mean age was 38 years and 58.9% was female (total sample). Participants were purchasers of nicotine patches, (adolescent) smokers, employees, visitors of websites.<br>[E] Relative Risk abstinence rates for Web-based-only interventions (1 email-based) RR 1.40 (CI 1.13-1.72).	High attrition and low statistical power may have led to insignificant results in the adolescent age group. Findings cannot be applied to smokeless tobacco users. The potential effectiveness for elderly, less educated, and those living in developing countries, is unknown.	Web- or computer-based interventions should be used as a key guideline recommendations for smoking cessation. More research with large sample sizes is needed to confirm the effectiveness of the smoking cessation interventions among adolescents or young adult smokers. Further research is needed to investigate program effects

Study characteristics	RE-AIM	Limitations	Recommendations
Total sample size: 12460/ 29549<br><br>Target group: Current smokers			for patients with specific conditions. Future developments should focus on standardized, high-quality web-based smoking cessation programs.<br>Although self-reports are less valid than biochemical measures, conclusions about effectiveness remain the same regardless of the method.
Shahab & McEwen (2009)<br><br>Study type: M<br><br>Articles: 11<br><br>Total sample size: 15511<br><br>Target group: Current smokers	[R] Studies targeted the general population, except for 2 studies (college students, adolescents). Participants were mainly white, female smokers with at least high school education of a wide age range (15.7-42).<br>[E] Five studies found an overall positive effect on smoking cessation rates. Three studies only found effects in sub-groups analyses, and three studies did not find a positive effect. A random effects model of studies that compared interactive, tailored internet smoking cessation interventions with minimal control conditions found a significant effect (RR 1.5 CI(1.2-1.9) Note: significant heterogeneity in effect size). The increase in quit rates was larger when interactive, online smoking cessation interventions were compared with a booklet/e-mail (RR1.8 CI(1.4-2.3)) than with a static website control condition (not significant). Pooled results from trials that did not require participants to intend to stop smoking in the near future ($n = 4$) indicate no intervention effect, but outcomes were heterogeneous. In contrast, while equally heterogeneous, the pooled results from trials recruiting smokers motivated to quit ($n = 7$) show a treatment effect (RR 1.3, 95% CI 1.0–1.7, $P = 0.04$).<br>A random effects model suggests that interactive, online interventions increased abstinence after 6 months follow-up by 17% (CI 12-21%) effectively doubling smoking cessation rates compared with minimal control conditions (RR1.9 CI(1.9-2.6)), based on point prevalence data.<br>[AIM] Website usage started off reasonably high, but decreased quickly over time. Participants of one study indicated their reasons for abandoning the intervention, e.g. stressful life events or the intrusiveness of the intervention. Support tools (discussion groups, chat rooms, 'ask the expert') were most popular opposed to educational material, one study found. Satisfaction with the program was measured in three studies and was found to be generally high (90%).	There is a lack of detailed description of interventions which complicated analysis. Few treatment moderators and mediators were examined explicitly in the reviewed studies. Biochemical outcome validation is impractical and unnecessary. The use of the internet may bias against people with low computer literacy. On the other hand, web-based interventions have shown to involve users commonly excluded from research.	It is important to balance user involvement and treatment complexity. Usability testing by potential users of online programs, rather than by experts, is likely to improve existing online interventions. The representativeness of trial participants needs to be established. Relatively small numbers and variable methodological quality limits generalizability. More needs to be done to improve reporting quality. Future research should evaluate the actual reach of the interventions. More research is needed to confirm the relative efficacy of interactive, online interventions compared with static websites and face-to-face counseling.
Civljak et al. (2010)	[R] Participants were mostly smokers motivated to quit	There is a small number of studies available. Most	More rigorous studies comparing the long-term effects of Internet

Study characteristics		*RE-AIM*	*Limitations*	*Recommendations*
Study type<br>Articles<br>Total sample size<br>Target group	M<br>20<br>33813<br>Smokers	smoking, who chose the internet as a tool for cessation support. Internet vs. non-internet/no intervention Studies recruited adults (n=6), young adults (n=1), adolescents (n=3). Participants were on average 16-57.4 of age and smoked on average between 4-21.8 cigarettes per day. Comparison between internet interventions Studies recruited participants of 18 years and older (n=7), of 21-70 years (n=1) and 2 studies made no age restrictions. Mean age ranged from 34-46.3. Participants smoked on average between 18.3 and 23.5 cigarettes per day and some studies required motivation to quit. There is some evidence that females are more interested in participating in online smoking cessation interventions.<br>[E] Internet vs. non-internet/no intervention Six of 10 studies found a significant effect of internet interventions compared to a non-internet intervention or no intervention.<br>Comparison between internet interventions Two of 10 studies found a significant difference between different internet interventions. This does not mean interventions were ineffective. Three studies showed positive results for tailoring, one only in depressed participants.<br>Two studies evaluated cost-effectiveness. One study calculated that the cost of running a clinic-intervention with 50 participants a month was equally expensive as running an internet intervention with 600,000 participants. Another study showed that less than 2000 dollars of money spent on programming gained 5000 new participants in an online intervention, compared to the costs of 100,000 dollar to be made for a telephone intervention reaching 1000 participants.<br>There is limited evidence to support Internet interventions on smoking cessation.<br>[AIM] Program use was the secondary outcome in 10 studies.	studies relied on self-reported data, but biomedical validation is considered to be unnecessary. Some large online studies had high loss to follow up rates. Assuming that those lost to follow up continued to smoke may introduce bias. Contamination in control groups may be difficult to prevent, because of unrestricted access to the Internet and it is unsure whether the intervention group is only using the intended intervention.	interventions with non-Internet interventions or no intervention at all are needed. These should assess outcomes using objective measures, and also assess cost-effectiveness considerations. Future trials should describe the likely mechanisms through which these interventions may (or may not) be exerting their effects – they should therefore also report data on patient satisfaction, changes in knowledge, motivation, dependency, quit attempts and safety considerations. Researchers should aim to assess smoking status after six months as a minimum, so that the longer-term benefit of programs can be determined and meta-analyses of outcomes across studies be facilitated.
Hutton et al. (2011)<br>Study type<br>Articles<br>Total sample	 SR<br>21<br>NR	[R] Studies were conducted among adults (n=15), college students (n=1), and adolescents (n=5).<br>Adults All trials were mixed sex (% males ranged from 29.5-84%). All but one had mostly Caucasians participants. Website-exposure: 7 studies reported on exposure, three reported a percentage ranging from 15.8-88%. In 6 of 7 studies higher quit rates were found among smokers who had greater website exposure. The number of web	The effect of web-based treatment is hard to estimate. Only half of the adult RCTs indicated to use theory and across trials behavior change methods varied widely. Web-based treatments are often combined with other treatments, complicating the effect evaluation. Information on participants was also limited; few studies examined differences by gender or socio-economic variables. Education showed some association with	Assessing barriers to participation in Web-based programs may improve uptake and efficacy of Internet-based interventions. Further investigation of theory, methods, and mode of delivery/assessment will advance understanding of effective strategies to help adolescents quit smoking. Future studies should focus on which treatment works best, for whom and under what conditions. RCTs are helpful in answering these questions. Interventions should define the theory that informs intervention design and systematically test which

Study characteristics		*RE-AIM*	*Limitations*	*Recommendations*
size<br><br>Target group	 <br>NR	sections opened was related to subsequent smoking cessation. Women opened significantly more treatment options than men.<br>College students Only one study addressed college students (n=517) and was aimed at occasional and light smokers.<br>[E] Adults<br>- Web-based interventions compared to other treatments (n=7) showed that intensive, multicomponent trials produce higher quit rates than self-help manuals. The effect of the web is hard to isolate. Web-based interventions appear more effective that no (delayed) interventions (n=2/7). Studies comparing web-based intervention to counseling (n=3/7) found no difference in quit rates.<br>- Web site-delivered interventions compared with other web site-delivered interventions were not shown to be more or less efficacious, evidence was insufficient. One study found that a more supportive website was associated with higher quit rates than a more informational one, two other studies found no effect. All studies had high retention rates.<br>The evidence for effectiveness of web-based smoking cessation program is modest.<br>College students The one study addressing college students showed that 30-day abstinence at 30-week follow-up was 40.5% in the multicomponent intervention group and 23% in the comparison group (p<.05). Biochemical validation showed weaker though still significant effects. The multicomponent nature of the study inhibits conclusions on web-based effectiveness for online elements could not be isolated.<br>Adolescents Evidence on the effectiveness of web-based interventions for adolescents is insufficient. Results were mixed.<br>[AIM] Overall, education showed some association with retention and abstinence.<br>Adults Retention rates ranged from 27-86%. In a study on tailoring, younger, male, or less formally educated participants were more likely to disengage from the program.<br>College students Retention rate was 92%.<br>Adolescents Retention rates ranged from 53-87%. In one study, lighter smokers, younger age, female and non-White participants were more likely to be abstinent.	retention and abstinence. Generalizability to developing non-White populations is unknown. Only a minority of studies incorporated or controlled for nicotine replacement therapy. A major limitation is the high loss to follow-up. Few studies reported on log-on data, making it unclear whether people failed to log on or did not benefit from the intervention. Just three trials offered incentives to encourage follow-up adherence. Intention-to-treat analysis was typically used to fill in missing data and missing participants were assumed to be smokers. Methodological heterogeneity limits evaluations of efficacy.	techniques within websites promote the greatest change in smoking cessation rates. It would be useful to examine exposure and dose for they are associated with higher quit rates.<br>It would also be useful to systematically assess the additive effect of supplemental modes of delivery and the use of non-trial cessation methods, especially pharmacotherapy. Nicotine Replacement Therapy may boost effectiveness of Web-based treatments. Future studies would advance evaluation of efficacy if length of treatment, type of treatment, and length of follow-up were more homogeneous across trials.<br>More information about the participants should be collected, like intention to change and whether someone is suffering from depression.<br>Future trials may adjust for baseline differences in participants, which could otherwise moderate treatment effects. Reducing attrition will continue to be a challenge. Problems of missing information may also be addressed in advance of data collection by more sophisticated statistical methods using multiple imputation techniques or other likelihood methods, such as random regression, mixed models or generalized estimating equations, survival analysis.

Study characteristics		*RE-AIM*	*Limitations*	*Recommendations*
Chen et al. (2012)		[R] Single tailored component<br>Participants were responders to invitations to participate in the trial advertised through various media. Mean age ranged from 37.9-46.3 years. One study focused on young adults. % female ranged from 45%-79%.<br>Multiple tailored component<br>Participants were either self-referred to smoking cessation websites or were responders to invitations to participate in the trial advertised through various media. Only current smokers were included in the analysis. Mean age ranged from 20-47.3 years. Three studies focused on young adults. % female ranged from 16-73%.<br>[E] Single tailored component Two of 6 single tailored interventions found an effect on smoking cessation; one of these only found an effect on depth of success stories and personalization of the source (e.g. using photographs). The other studies found no significant effect. Pooled estimate of point prevalence abstinence based on short/term follow/up (<3 months) favors the web-based interventions (significance was reached with a fixed effect model, but not with a random effects model).<br>Multiple tailored component Four of 12 studies found a significant effect, one showed a short-lived effect (at 3 months, but not at 6 months) for a web-based intervention supported by phone-counseling and pharmacotherapy, one showed an ineffective modification of and effective online intervention, one showed that a tailored version of the intervention improved smoking cessation and the last showed that a tailored web-based intervention was more effective than untailored e-mails.	The review only focuses on adult smoking. Only a small number of factors that could potentially influence the effectiveness of the interventions were investigated.	Further research is needed on the relative benefits of different forms of delivery for electronic aids (internet, mobile telephone) and the content of delivery (including more research on the efficacy of interactive electronic aids). There is a need for further research on the acceptability of technologies for smoking cessation with subpopulations of smokers, younger or older smokers. Research with poorer smokers, who increasingly constitute the largest group of smokers in developed countries, is required now. Research gaps exist relating to the usability and acceptability of electronic aids for smoking cessation in particular settings, like secondary care. More evidence is required on the relationship between involving users in the design of interventions and the impact this has on effectiveness, and on how electronic aids developed and tested in research settings are applied in routine practice and in the community. Research should be done on the potential for electronic aids to help prevent relapse to smoking either as an adjunct to face-to-face or telephone behavioral support or as part of a longer term stand-alone intervention.
Study type	M			
Articles	18/60			
Total sample size	32425/ NR			
Target group	Adult smokers			
CONDOM USE				
Noar et al. (2009)		[R] The three internet interventions reached young adults (mean age 21.75, 53% men), Dutch men who have sex with men (mean age 33, 100% men), and heterosexual adolescents (mean age 15.59, 56% women).<br>[E] Effect sizes for condom use were 0.095 (95% CI -0.030–0.220), 0.284 (95% CI 0.088–0.480), and 0.200 (95% CI -0.222–0.622). Overall analyses showed that interventions were significantly more efficacious when they used individualized tailoring and a Stages of Change model.<br>[AIM] Two trials with poor retention compared to the computer-based interventions were also the two Internet-		Given that the Internet has emerged as a conduit for individuals to seek and find high-risk sexual partners, developing and testing interventions that can proactively reach out to such populations is a high priority. Thus, future research to improve ways of conducting randomized trials online is warranted.
Study type	M			
Articles	3/12			
Total sample size	1638/4639			
Target	Individuals of HIV-			

Study characteristics		*RE-AIM*	*Limitations*	*Recommendations*
group	negative or unknown serostatus	based trials (the third internet intervention recruited individuals in schools).		
		WEIGHT MANAGEMENT		
Weinstein (2006)<br><br>Study type<br><br>Articles<br><br>Total sample size<br><br>Target group	 <br>SR<br><br>7/8<br><br>690/794<br><br>Overweight or obese adults (>30 years of age)	[R] Weight loss Participants were mostly women (74-100%), except for an intervention in a military setting were 100% was male. Mean BMI ranged from 26.4-33.1.<br>Weight loss maintenance Participants were mostly (white) women (80-85%). Mean BMI ranged from 31.8-33.7.<br>[E] Weight loss All studies (n=7) showed a significant positive effect on weight loss, except for one commercial program. Internet interventions that included a behavioral therapy component (n=2) showed greater weight loss. Internet interventions are effective, but may be no more effective in producing weight loss than traditional face-to-face weight loss programs are.<br>Weight loss maintenance Three studies showed no favorable effect for the internet intervention compared to face-to-face contact. One study showed that the internet group gained more weight and earlier than the control groups. These three studies conclude that internet weight loss maintenance interventions have a limited appeal to participants and that face-to-face contact seems more promising.	Women outnumbered men more than two to one, therefore results are difficult to generalize to men. Participants in the reviewed studies were predominantly white with more than a high school education.	Any intervention that can personalize the Internet experience, such as face-to-face encounters with the health professionals who will be facilitating the online communications, may increase attendance.<br>More studies that include large numbers of men are needed. Program design may play an important role in gender appeal and is worth examining.<br>The Internet may have great appeal for individuals under the age of 30, it warrants further investigation on this population.<br>Accessibility is rising due to the narrowing of the digital divide; suitability of the intervention deserves attention now.<br>Internet-based weight loss programs should be designed so that they are consistent with the user's needs, values, and social and cultural patterns and operate at a level of complexity that is understandable. Following such guidelines will allow researchers to systematically evaluate and determine where difficulties in adoption occur.<br>Individually tailored programs that consider the user's needs, motivations, values and constraints should improve the chances of success.
Saperstein et al. (2007)<br><br>Study type<br><br>Articles<br><br>Total sample size<br><br>Target group	 <br>SR<br><br>6<br><br>NR<br><br>Adults	[E] Five of the six studies showed that weight loss programs could be effectively delivered over the Internet. Personalization through ongoing tailored information and feedback, either via mail from a human counselor or a computer-based program, was a critical component. Control groups consisting of Internet-based programs primarily delivering information and offering general online support were not effective in helping participants lose weight. The one study that appeared ineffective in weight loss lacked the structured approach of the manual used by the control group. In another study, an e-counseling component (providing feedback, support reinforcement and recommendations for change) was isolated to be effective. The findings of these studies showed that Internet-based tools could help users lose weight if they provided a structured, personalized program with an emphasis on diet, physical activity and cognitive-behavioral strategies.	With multicomponent interventions it is hard to tease out which components or combination of components led to the study outcomes.<br>Distribution of effective interventions is limited and readily available interventions with unknown effectiveness are abundant.	Objective physical measurements, like in-person weighing, may increase participant's sense of responsibility or accountability towards the study.<br>Continued emphasis on helping consumers develop the ability to evaluate websites is important, and we need to continue educating the public about research findings regarding effective online strategies and programs.<br>Along with continued development and refinement of online programs, it is needed to determine methods for increasing the distribution of programs with known efficacy.<br>Future research needs to focus on understanding who may be best served with online weight loss programs, gauging the applicability of programs for more diverse audiences.

Study characteristics		*RE-AIM*	*Limitations*	*Recommendations*
		[AIM] Several studies showed that those who logged in more frequently also lost more weight.		
Maon et al. (2012)<br>Study type<br>Articles<br>Total sample size<br>Target group	 M<br>30<br>12500<br>Healthy population	[R] 30% of total participants were children and adolescents. 67% of studies were conducted in adult populations, recruitment occurred through workplaces, academic institutions, and in community settings.<br>[E] 93% of the studies demonstrated the effectiveness of web-based interventions to motivate participants to engage in healthy eating and active living, however, nine studies did not show significant differences between intervention and control groups.<br><u>Dietary practices</u> Four of eight studies showed significant effects on increase of knowledge, intention to change, awareness and self-efficacy in relation to healthy eating practices (e.g. increased fruit or vegetable consumption).<br><u>Physical activity</u> Seven of thirteen studies showed significant effects on physical activity levels, such as increased walking or decreased sedentary behavior.<br><u>Combination</u> Seven of nine studies showed significant results on targeted behavior, i.e. in healthy eating and exercise.<br>Positive outcomes were also associated with interactive interventions (e.g. emails, discussion boards, chat sessions, etc.). Theory-based interventions were also effective. The effects of the interventions declined as the length of time from the end of the intervention increased.	The effects of web-based interventions are short-lived. This is probably due to the problem of engaging and retaining people to use websites.	E-mail communication is a useful tool to increase the rate of repeated participation in websites. More studies are required to investigate strategies to engage and retain people in using websites. Mobile technologies and web 2.0 technologies (social networking) may be important. Few studies have investigated the effectiveness of web-based technologies for the benefit of children and adolescents. Web-design also needs further investigation.
Fry et al. (2009)<br>Study type<br>Articles<br>Total sample size<br>Target group	 SR<br>12/19<br>11239/ 15655<br>NR	[R] Approximately 65% of all subjects were women.<br>[E] Of twelve relevant studies, all found positive effects. Studies focused on physical activity (n=3), weight loss (n=5), weight loss maintenance (n=1), nutrition (n=2), and both physical activity and (n=1). Tailoring was used in ten out of twelve studies.<br>[AIM] Five studies reported on associations between the level of interaction participants had with the intervention and outcomes and these were all positive. 11 of 19 articles reported positive findings regarding the utility of periodic prompts.	It is difficult to assess the value of individual intervention elements. There is a lack of follow-up data, and the data available is heterogeneous. Participants were volunteers and therefore a more motivated sample might be reached. There was a relatively high proportion of females in the samples. Men in the samples may not be representative for the general male population. Collecting the data face-to-face might also be a biasing factor.	Additional research on limited contact intervention is warranted. It would be valuable for future studies to use no-treatment control groups, include long-term follow-up data collection, and test specific intervention components or prompt characteristics instead of entire programs. Further investigation into the effectiveness of different time intervals between prompts would be highly valuable. It would also be informative if researchers were able to include a more representative proportion of men in studies to see if they respond differently to these types of interventions. Further research comparing reach and effectiveness of various types of communication technology is recommended.
An et al. (2009)<br>Study type	 SR	[R] Study participants ranged in age from 8 to 18 years and included both boys and girls.<br>[E] Six of eight studies (75%) reported that Internet	In an intent-to-treat analysis, high dropout/attrition rates affect the ability to detect small differences between the groups.	To reduce high dropout rates resulting from the nature of Internet interventions, a run-in period may be employed. Requiring users to visit the intervention website prior to enrollment and randomization,

Study characteristics		*RE-AIM*	*Limitations*	*Recommendations*
Articles	8	interventions, either as stand-alone programs or as combined interventions with other behavioral approaches, demonstrated clinically meaningful as well as statistically significant results in terms of clinical effectiveness on the measurable outcomes such as BMI, weight loss, physical activity, and dietary fat intake.<br>[AIM] Attrition rates reported for each study ranged from 4.9-30%.	Internet interventions may not be as appropriate for underserved populations.<br>All of the studies included in this review were conducted in the United States, restricting generalizability.	the possibility of dropouts during the intervention period would be reduced.<br>Previous research has suggested that social cognitive theory may be a theoretical base for developing personalized and tailored Internet interventions for behavior change and weight loss. It is also recommended that increased frequency of personal contact and the amount of interaction, referred to as dosage or interactivity, should be considered a key element for successful implementation of Internet interventions.<br>Children require guidance as well as encouragement and support as they transition through the stages and processes of development.<br>Future research should increase rigorous methodological adequacies such as adequate sample sizes, investigate types of interventions for the best match with children and adolescents, include accurately measurable outcomes, and examine cost as well as clinical effectiveness of the intervention. It is suggested that researchers incorporate interactive and personalized components with up-to-date information to make longer interventions available for evaluating long-term effect on weight loss.<br>Future research should recruit male and female study participants from diverse racial/ethnic and cultural backgrounds, varying levels of socioeconomic status, and different geographic locations both within the United States and globally.<br>Identifying a theoretical framework for Internet-based interventions is an important area for future research. Standardized study frameworks including behavioral protocols linked to specific conceptual and operational definitions of the theoretical-based intervention should be emphasized.
Total sample size	3697			
Target group	Children and adolescents			
Neve et al. (2010)		[R] 77% of participants were female (3.7% unclear).<br>[E] Weight loss n=13 Three studies showed effectiveness, defined as ≥5% weight change. Significant difference between groups was found in 10 studies, of which one favored the control group.<br>Weight loss maintenance n=5 One study showed effectiveness, defined as ≥5% weight change. Significant difference between groups was found in 2 studies, of which one favored the control group.<br>Ten studies measured participants' usage of self-monitoring tools in the form of online diaries to record weight, food and drinks consumed and/or physical activity undertaken. Five weight loss intervention studies and two weight loss maintenance studies explored correlations between weight change and level of self-monitoring with six studies reporting a significant correlation between the	The majority of studies did not report percentage weight change. Therefore, we cannot determine the true number of effective web-based interventions. We also cannot conclude absolutely that there is no difference in weight loss between web-based and control groups due to the heterogeneity identified in the meta- analysis.<br>Little research is done on personalized feedback or tailored information. The intervention length of four studies was less than the standard behavioral treatment intervention length of 16 weeks.<br>The long-term impact of web-based programs remains unknown due to the lack of follow-up beyond the intervention.	No studies were identified that explored the effect of web-based interventions with enhanced behavioral features on weight loss maintenance; therefore research is also warranted in this area.<br>It is recommended that future RCTs explore strategies to improve retention rates, ensure interventions are of an appropriate length and follow-up participants for at least 1 year post intervention to increase the likelihood of detecting weight and behavior change.<br>Future RCTs or longitudinal studies should report usage as a proportion of possible or expected contacts with the particular web-feature and aim to ascertain the ultimate dose required to achieve satisfactory weight loss or maintenance, while maintaining adequate attrition rates.<br>There is a need for succinct research questions and detailed intervention descriptions to allow readers to truly understand the components of the interventions being compared. It is also important to interpret the meta-analysis results with caution due to this
Study type	M			
Articles	18			
Total sample size	5700			
Target group	Overweight adults			

Study characteristics	*RE-AIM*	*Limitations*	*Recommendations*
	two. Four studies explored the level of peer/social support of which 3 showed a positive effect for the web-based interventions. Three studies demonstrated higher attendance levels in face-to-face groups compared with a web-based group. Two studies demonstrated a significant correlation between attendance at group meetings and weight change.<br>[AIM] Four studies reported statistically significant differences in number of log-ins between groups, three of which demonstrated higher log-ins in a web-based intervention with behavioral therapy. Seven studies investigated associations between weight loss and number of log-ins, with five studies showing a greater number of log-ins was associated with increased weight loss. One study explored associations between number of log-ins and attrition, and found that the initial number of log-ins were significantly lower among those who dropped out by 12 months compared with those that completed the intervention.<br>Retention rates varied from 48 to 100% at the post-intervention time point. Eight studies retained greater than 80% of participants, five retained 60–80% and two 40–60%, one did not provide retention rates for the end of the intervention, and in one intervention length varied.		additional potential source of heterogeneity.<br>There is a need for a consistent definition of effective weight loss and weight loss maintenance to be adopted by researchers.<br>Such a definition would allow for greater comparability between studies and meta-analysis of results. Future research in the area should prioritize well-designed efficacy trials comparing web-based interventions with the traditional methods of delivering lifestyle interventions (e.g. individual and group-based counseling) or to waiting list controls. Studies should be designed to determine which components of web-based interventions are critical to achieving efficacious weight loss and/or weight loss maintenance, including determination of an optimal usage or intervention dose. To ensure high-quality research in the area, strategies to improve retention rates and engagement with the web-based intervention (e.g. through email reminders, telephone prompts or via enhanced program features) should be further explored. In order to facilitate future systematic reviews and meta-analyses of program effectiveness all studies should report participants mean percentage weight change. To address the gap in long-term follow-up of participants' studies need to report results up to at least 1 year post intervention and ideally longer.
Manzoni et al. (2011)<br>Study type: SR<br>Articles: 26<br>Total sample size: 8324<br>Target group: Overweight / Obese adults	[R] At least 76.7% of all participants were female. Mean age is 46.2 years. Eighteen interventions recruited participants from community, three interventions through health care system, two studies recruited participants on work sites, one study among university students and one from US Air force.<br>[E] <u>Weight loss</u> Behavioral internet-based weight-loss interventions enhanced by professional feedback provided through internet is more effective that a web-site only program. Internet-based interventions achieve similar weight loss to minimal intervention groups.<br><u>Weight loss maintenance</u> Apart from one study showing no further reduction in weight after completion of a maintenance program following a weight-loss intervention and no significant difference between the groups, all the other studies showed a further improvement in mean weight loss for both groups. Evidence suggests that internet-based interventions achieve similar levels of weight loss maintenance to face-to-face interventions, and less weight is regained in comparison with no	Studies are very heterogeneous and any attempt to compare the results must be viewed with caution. No study has compared an internet-based program with a "real" control group. No study has directly compared an internet-based intervention with a traditional individual or group-based behavioral treatment. Only two studies out of the 26 reviewed in this paper assessed cost-effectiveness of an internet-based intervention. No study examined gender differences and generalization of results to men is questionable. A standard method to evaluate adherence was not used and no meta-regression can thus be performed to examine that association in a meta-analytical fashion. No studies have been done with obese patients referred to specialty clinics or to bariatric surgery.	Future controlled randomized trials should report compliance as a proportion of recorded to expected contacts with the particular web-feature. Future studies should adopt one of the methods to account for noncompliance. Among the studies included in the present review, one only used such a method, i.e. per-protocol analysis.<br>Future research should prioritize well-designed efficacy trials that account for known sources of variation and determine which features of internet-based interventions are critical to achieve success in weight loss and maintenance in a cost-effective and time-saving manner. Future research should also develop and evaluate internet-based weight-loss interventions that are specifically tailored to the needs of the health-care delivery system.

Study characteristics	RE-AIM	Limitations	Recommendations
	intervention.<br>At present, the only factor that has received some empirical support for being a mechanisms or mediator that leads to weight loss and maintenance, is e-mail contact with a professional counselor which provides feedback and reinforcement along the intervention.<br>[AIM] Attrition rates varied from 0-70% at the post-intervention time point.		
Aalbers et al. (2011)<br>Study type: SR<br>Articles: 7/12<br>Total sample size: 2444/4984<br>Target group: People aged 50 and older	[R] Overall mean age was 54.9 (±8.3) and 62.2% of participants were female. Two studies reported highly educated study populations and none reported lower educated populations compared to the general US population.<br>[E] Five studies permitted effect size calculation. Effect sizes range from -0.30 (online generic newsletter had more effect than online tailored newsletter) to 0.82, but on average the effect sizes are small to moderate-small. Complex interventions, whether they present tailored or generic information, and online or offline comparison, are more effective than interventions with only one component.<br>[AIM] On average the interventions had an attrition rate of 18.3%. One study measured dose-response relationships and found a significant interaction effect in maintaining increased total physical activity values between control and intervention. The intervention group had significantly more exposure ($p < 0.01$; 8.2 versus 2.8 total logins) to their website than the control group. Within the intervention group, users maintained their increased use of the community walking path significantly better than the non-users (59.2 versus 25.0 in/week respectively, $p = 0.05$). Tailored information seems to lower attrition rates, but data was limited. A self-monitoring tool was used in six studies, amongst others with the purpose to decrease attrition. The median attrition in these six studies was below the median attrition for the other components. Two of five studies reporting on the use of online goal setting studies found sustained usage at the last follow-up measurement. Tailored information seems to lower attrition rates, but data was limited. One study found that tailored interventions were rated as more interesting (80% positive comments versus 38% in the generic group, $p = 0.01$), and helped subjects to become more active (44%	Social network forums were used in more than half of the studies reported by this review. All reports on the usage of these forums pointed towards low uptake and activity rates. Goal setting activity decreased over time in all studies. Most importantly, the small amount of articles in this area makes it hard to draw generalized conclusions. The spread on focus areas and the fact that some studies compare online groups with online control groups, while other studies compare online groups with offline control groups, makes comparison difficult and meta-analysis impossible. All study populations, in the current review, except for one, are specific populations unrepresentative of the general population the decision to limit the literature search to articles published in English and Dutch may have led to selection bias.	In a setting where complex interventions are used it is vital to document and evaluate how components work, are used, and influence the outcome. Process data about how frequent the tool is used, for what period of time, by which subgroups, and for what reason should standard be provided. A qualitative participant-oriented approach should be used, questioning participants which components they felt were effective in hanging their lifestyle. Goal setting is an effective way to facilitate lifestyle behavior change in people above 50 years of age, and self-monitoring is a core behavioral intervention component in weight loss and physical activity interventions.<br>If these components are used, it is advisable to clearly state the goal of both components and the added value that they can have for achieving lifestyle change. Using email notification systems and generic or tailored feedback via email could provide the opportunity to engage people in the intervention and remind participants of their behavior change goals and progress.<br>We need more data on the dose-effect relation, i.e. the effect of intervention exposure on intervention effectiveness. In all studies identified in this review participants were recruited offline. Online recruitment strategies should be developed additionally. Possibilities lie in internet advertisement, optimizing a website to gain a large amount of hits on search engines, web links on other health related, national health associations and governmental agencies-websites or sending recruitment emails to all employees of large corporations. Researchers of Internet-mediated interventions should take precautions to recruit an equal number of lower educated people and men into their intervention to avoid selection bias. Social networking forums are frequently used as a tool but rarely used by the participants, and thus are ineffective. Time may have come to incorporate modern features (i.e. web 2.0) into online lifestyle interventions.

Study characteristics	RE-AIM	Limitations	Recommendations
	versus 24% in the generic group, p = 0.001). However, the most interactive features were still used the least.		
Kodama et al. (2012)<br><br>Study type: M<br><br>Articles: 23<br><br>Total sample size: 8697<br><br>Target group: Overweight or obese adults	[R] In about half of the included studies (11 studies), participants were women-dominant (≥80%). Study-level mean (±SD) age and BMI were 46 (±6) years and 32 (±3) kg/m^2, respectively.<br>[E] Overall, using the Internet had a modest but significant additional weight-loss effect when compared with results in non-Web-user control groups (-0.68 kg, *P*=0.03). However, large and highly significant between-study heterogeneity was observed in the effect size (I^2 = 84.4%; *P*<0.001).<br>In comparison with control groups, although a significantly favorable effect was observed when Internet support was added to the experimental group as an adjunct to the obesity care provided to all participants in an individual study (net effect (95% CI) (kg),-1.00 (-1.57 to -0.43), *P*=0.003), an adverse effect was observed when the Internet was provided to the experimental group as a substitute for the face-to-face support similar to that given to the control group (net effect (95% CI) (kg), +1.27 (0.29–2.25), *P*=0.01). Although adding face-to-face support to Web-based support enhanced the weight-loss effect of using the Internet (net effect (95% CI) (kg), -1.93 (-2.71 to -1.15), *P*<0.001), the weight-loss effect was non-significant when face-to-face support was not provided (net effect (95% CI) (kg), -0.19 (-0.87 to 0.49), *P*=0.59). The difference between results with and without face-to-face support was significant (P=0.003).<br>In comparison with the non-Web user control group, the Web-based program was effective when the aim of using the Internet was initial weight loss (n=18) (net effect (95% CI) (kg), -1.01 (-1.68 to -0.34), *P*=0.03), but was ineffective when the aim was weight maintenance (n=5) (net effect (95% CI) (kg), 0.68 (-0.50 to 0.85), *P*=0.26).<br>The additional weight-loss effect in online weight-control intervention studies was attenuated with a longer educational period. Additionally, the Web-based program was found not to be superior to other obesity treatment programs when its aim was to maintain body weight after a previous weight reduction.<br>Of content elements, only individualized instructions on the Website being effective (net effect (95% CI) (kg), -1.33 (-2.32 to -0.34), *P*=0.008).	One of the major problems in using the Internet for weight loss appears to be long-term sustainability of the Internet program. Internet users may acquire 'tolerance' to stimuli that text messages or e-mail communications give in the course of the Web-based educational program and become bored with these stimuli. This would make it difficult for participants to maintain their motivation for lifestyle modifications, which may diminish chances for success in weight control.<br>This analysis might not necessarily provide clinically sufficient evidence for the long-term effect of an Internet component on weight control. Employing a double-blinded method is impossible in the used RCTs. The participants assigned to the control group might have felt a sense of competition with the Internet group, which could have created bias toward weakening the effect of the Web-based intervention. Poor compliance is recognized as a major issue in most online studies. Few studies have been conducted on Web-based weight-loss programs targeted exclusively for men, partly because of the fact that men are less interested in weight loss than women.	An in-person contact approach is superior to a technology-based approach from the viewpoint of the amount of weight loss; if used, an Internet program needs to include the component of a face-to-face program for participants to achieve weight loss. It is necessary that Web-based obesity-care programs become more widespread and are developed to result in greater weight-loss effect than the present Web-based systems.<br>It is suggested that Web-based lifestyle modifications should gradually become more intensive or should be combined with in-person support, especially when the aim is success in maintaining long-term weight loss.<br>A key to improve the efficacy of Web-based weight-loss programs might be the development of Web-based instruction rather than self-monitoring or communication via the internet. To make the instruction more attractive, the text messages should not be repetitious but novel and more thoughtful of the personalized aspects of behavioral therapy as if the Web-users would consider the automated messages the same as if they were given face-to-face.<br>Future research should address (1) a cost-to-benefit ratio that involves, in addition to the amount of weight loss, the reduction in incidence and medical cost of obesity-related diseases, such as hypertension, diabetes, and furthermore, cardiovascular diseases as a benefit of obesity care; (2) the proper proportion of online and in-person obesity care support for maximizing the cost-to-benefit; and (3) the characteristics of obesity care (for example, standardized outpatient practice or self-help material) or of obese persons (for example, computer experience, health literacy and type of occupation) that can benefit from supplemental care using the Internet.<br>The Internet effect in obesity treatment appears to depend on the usage of the Internet or the period of its use, rather than on participants' characteristics such as age or gender. Although an in-person approach is suggested to have a greater weight-loss effect than a Web-based approach, the development of technology-based programs should be continued from the viewpoint of saving time and cost, especially focusing on appropriate combinations of face-to-face and Web-based support, as well as making educational programs more attractive for participants.

Study characteristics	*RE-AIM*	*Limitations*	*Recommendations*
	[AIM] Intervention periods, including both educational and observational periods, ranged from 3–30 months. Mean (range) of the dropout rate was 17.8% (0–79.6%).		
Coons et al. (2012)<br>Study type: SR<br>Articles: 8/13<br>Total sample size: 2604/3081<br>Target group: Adults	[R] Three studies were all-male, in the remaining studies the percentage females ranged from 33.0-93.0%. Mean age ranged from 35.9 (±11.1) to 46.6 (±9.9) years.<br>[E] Four of eight weight loss trials reported significantly greater weight loss among individuals randomized to internet interventions compared to controls. One trial analyzed only participants with complete data and another lost 43% of their sample due to attrition. Components of successful interventions include self-monitoring, goal setting, and providing feedback on current diet and activity behaviors relative to daily and weekly goals. Three trials reported no significant difference in weight loss between intervention and control conditions, all of these trials reported within-group weight loss. One trial reported significantly greater weight loss in the control condition (i.e. face-to-face group weight loss) compared to the intervention conditions (i.e. Internet alone or hybrid conditions).<br>[AIM] Retention rates ranged from 57-96%. Three studies reported a comparison of adherence between groups. Two of these studies reported no significant differences between groups on adherence measures. One study reported better adherence in the comparison group (e.g. 34% of participants completed all sessions in the phone intervention group versus 18% in the technology group). In two trials, only reporting adherence data within the Internet group, adherence to the web-based interventions was poor, ranging from 28% to 41.2%. Only one study reported any data on participant satisfaction and usability of the technology; participants reported that they were highly satisfied with the website, finding it to be enjoyable and usable.	Five of the trials reported attrition rates >20% (ranging from 25% to 43%), which compromises the validity of the trial outcomes and power to detect significant between group differences.<br>Two of the trials included in this review conducted completer analyses, rather than employing the intent-to-treat principle. Although this practice lends insight into the effect of the intervention on those who complete it, this undermines the effect of randomization, limiting the conclusions that can be drawn. Few trials paid adequate attention to adherence to intervention components.	Efforts to streamline the self-monitoring process may promote adherence to well-established weight loss behaviors. More studies enrolling diverse samples are needed to verify the results of positive trials. Self-monitoring of dietary intake still relies on self-report. Consequently, efforts to develop objective measures of dietary intake will provide the most accurate information to ensure fidelity to weight loss interventions.<br>Future interventions should include self-monitoring (of diet, physical activity, and weight), goal setting (to reduce calorie intake and increase calorie expenditure), feedback on weight loss behaviors, and social support from coaches or peers. Clinicians looking to implement or recommend technology-enhanced interventions to their overweight and obese patients should ensure that programs include these established treatment components.
Norman et al. (2007)<br>Study type: SR<br>Articles: 19/47<br>Total: 2730/13299	[R] Physical activity (sample size: 1085/2364) Four studies focused on adults, one on children. All samples had more women than men, ranging from 51-100% females. Mean age ranged from 24.8-56.1 in the adult interventions. The children were fifth-graders.<br>Dietary behavior (sample size: 291/5384) Two studies focused on adults (mean age 40-49.6), one on children	Many studies suffered from low dose and poor utilization with a majority of participants failing to engage in more than half of the expected eHealth activities or had few website log-ons. For web-based interventions, log-on rates tended to decrease over time. In studies where dosing information was available, the data suggested that most participants received inadequate doses.	Methods are needed to motivate participants to use and reuse eHealth programs, so that optimal intervention doses are received by participants. Alternatively, more engaging, dynamic website programs may help keep participants engaged. It may be that eHealth programs are optimal for implementing certain intervention tasks (e.g., such as conducting assessments and providing an information resource), which can then give health professionals more time to help patients

Study characteristics		*RE-AIM*	*Limitations*	*Recommendations*
sample size<br><br>Target group	 <br>NR	(12-16 years). One study was 100% female, one 60% and in the school-bases sample 52% female.<br>Combined intervention (sample size: 1354/5551)<br>Seven studies focused on adults, four on children. Most samples were predominantly female, except one all-male study in Air Force men. Three studies were all-female and the remaining studies ranged from 49-89%. Mean age ranged from 20.2-46.3 years in adults and children were 8-15 years of age.<br>[E] Physical activity n=5/13 Only one of the studies showed findings favoring the internet intervention. The remaining studies, findings were statistically indeterminate. Of intervention which isolated the effect of internet, effect size estimates ranged from 0.02-0.31. Three studies found no significant effect size for physical activity.<br>Dietary behavior n=3/16 One study found significant changes favoring the intervention group on fruit and vegetable intake, saturated fatty acid ratio, and HDL cholesterol. Another study found no differences and one study had findings that favored weekly in-person meetings compared to an internet-based intervention. In an intervention which isolated the effects of the internet, effect size estimates on dietary fat intake ranged from 0.24-0.28. Effect sizes for fruit and vegetable intake ranged from 0.05-0.15.<br>Combined intervention n=11/20 Of the studies measuring physical activity, three favored internet interventions for increasing physical activity. Of the studies that measured dietary behaviors, 4 favored internet interventions for changing dietary behaviors. Of the studies that measured weight change, two studies favored internet interventions, and two studies found internet interventions to be less effective for weight loss compared to an in-person therapist and a standardized weight loss manual. Six of the eleven studies found evidence in favor of internet interventions on at least one of three outcomes of physical activity, dietary behavior, or weight loss. Of intervention which isolated the effect of internet, effect size estimates on weight loss were 0.19.<br>[AIM] Studies with higher utilization and dose tended to have better behavior change outcomes. Participants had higher utilization of behavior change websites compared to educational or control websites in several studies.<br>Physical activity Retention rates ranged from 73.8-96.8%.	The design of many studies precluded tests to determine whether the interventions were working through hypothesized theoretical constructs. As a result, when an intervention program resulted in weak findings, conclusions could not be drawn as to whether the lack of findings was due to a lack of theoretical fidelity or to other threats to study validity.	with problem-solving and information synthesis.<br>More research is needed to better determine how technology can be incorporated into programs to enhance behavior change outcomes.

Study characteristics	RE-AIM	Limitations	Recommendations
	Dietary behavior Retention rates were 77.8 and 94.9%, unknown in the school-based sample.<br>Combined intervention Retention rates ranged from 38.1-93%.		
**SUBSTANCE USE**			
Champion et al. (2012)<br>Study type: SR<br>Articles: 7/12<br>Total sample size: 8502/ 20633<br>Target group: Highs school students	[R] All trials were mixed gender and most targeted students in their first two years of high school (13–15 years of age).<br>[E] Tobacco Three trials targeted tobacco only and two were associated with some reduction in smoking. In one trial, there was only a small effect at post-intervention and the other intervention was only effective at reducing cigarette use among nonsmokers at baseline.<br>Alcohol All three trials that measured alcohol consumption were associated with some reduction in alcohol use at post-intervention and/or follow up. Effect size (ES) was small at post intervention (ES 0.09) and similarly modest at follow up (ES 0.16–0.38 and odds ratio 0.36–0.71). One trial was associated with positive outcomes relating to the frequency of binge drinking. Effect size and odds ratios for drug and alcohol use were small. Eight trials collected data post intervention and the follow-up period in the studies ranged from 6 to 34 months. Of the 6 trials that assessed drug and alcohol consumption at follow up, 5 showed lasting effects, ranging from 6 months to 34 months in length.	The trials included in the review relied solely on student self-report. However, studies have found the self-report of behaviors such as substance use among adolescents is highly consistent with behavioral observations, as long as confidentiality and anonymity is assured.<br>A further limitation is the small number of studies included in the review, differences in outcome measures assessed and the unavailability of data to calculate effect sizes for three of the identified programs. Only two of the 10 programs had been evaluated more than once. Of the 12 trials included in this review, only two analyzed results separately for males and females, and only one of these had available data to calculate effect size.	As well as establishing whether Internet-based programs are efficacious in preventing alcohol and drug use, it is critical to gauge why these programs might be having an effect. One factor that may be associated with program success is the number of sessions included in the intervention. An additional factor that has been cited previously as contributing to program success is the inclusion of booster sessions. All three programs that included booster lessons showed significant effects. Some of the effective ingredients in Internet-based prevention programs are normative education, resistance skills training and reducing positive expectancies. It is possible that teenagers may have realistic positive expectancies about alcohol or drug use, and may actively desire to alter their conscious state. Future prevention programs that address these potential motivations, in addition to social influence factors, may produce larger intervention effects for adolescents. Future trials may benefit from adding a parental component to existing Internet-based programs for drug and alcohol prevention. There is a need for cross-validation and replication studies of these existing programs, to provide further support for the effectiveness of Internet-based prevention for alcohol and other drugs, delivered in schools. Where sample size and power are adequate to do so, future evaluations should attempt to consider results for males and females separately, especially in countries where the recommended drinking guidelines differ for males and females.
Lehto et al. (2011)<br>Study type: SR<br>Articles: 21/23<br>Total sample size: 28483/ 33529<br>Target group: NR	[R] Of the interventions addressing smoking, 10 were aimed at adolescents, 3 at college students, 2 at adolescents, and 1 at sixth to ninth graders. Of the alcohol interventions 4 were aimed at adult (problem) drinkers and 1 was aimed at college freshmen.<br>[E] Twelve of 21 studies found a positive effect on behavioral outcomes. 9 of these addressed smoking prevention (n=16) and three problem drinking (n=5).		The presentation of detailed information about the theoretical basis, functionality, content, and structure of a Web-based intervention helps to interpret the results and conduct evaluations as on a more finely grained level.<br>Persuasiveness of an intervention component/application/system is a more complex issue and has yet to be tackled in future endeavors.<br>In order for widespread adoption, dissemination, and extended use of technology-enabled health behavior change interventions to take place, it is necessary to investigate not only how the interventions affect individuals, but also how individuals interact with technology and each other. Further research is also warranted to increase our understanding of how and under what circumstances specific persuasive features (either in isolation or collectively) lead to positive

Study characteristics	RE-AIM	Limitations	Recommendations
			health outcomes in Web-based health behavior change interventions across diverse contexts and populations.
Rooke et al. (2010)<br>Study type: M<br>Articles: 32/43<br>Total sample size n=: 9438/ 10632<br>Target group: NR	[R] 19 studies targeted young adults, 9 targeted adults (30+), 1 study targeted adolescents and three did not report age. Three studies were all-female, 3 all-male and the gender of the remainder of studies was mixed.<br>[E] Of all 32 studies effect sizes were calculated. Three were negative (-0.04, -0.14, and -.017), the rest ranged from 0.01 to 0.72. According to the conventions recommended by Cohen, where a *d* of 0.20 is categorized as small, 0.50 as medium and 0.80 as a large effect size, 15 studies had a small effect size, 11 were medium and 3 had large effect sizes. Moderator analyses revealed that web-based interventions (*d*=0.18, CI 0.10-0.25) were significantly less effective than offline formatted interventions (*d*=0.27, CI 0.14-0.41).		Minimal-contact interventions that have fewer sessions and can be accessed from home may be as effective as high-cost, labor-intensive interventions. Computer-delivered programs could make a valuable contribution to primary and secondary substance use prevention efforts aimed at adolescents. Future research could explore this possibility further.<br>Additional research focusing upon adolescents is needed.
Tait et al. (2010)<br>Study type: M<br>Articles: 16<br>Total sample size: 10532<br>Target group: Adolescents and young adults	[R] 14 studies were aimed at young adults, and two studies targeted adolescents.<br>[E] Young adults All studies were aimed at alcohol prevention. The overall effect size for the outcomes was *d*=− 0.22 (SE, 0.06; 95% CI, − 0.34 to − 0.10), but with significant heterogeneity (Q = 249.03, df=55; P < 0.00001). Those who received the interventions had a lower level of alcohol consumption at follow-up than those in the control groups, with a mean difference of d = − 0.12 (SE, 0.05; 95% CI, − 0.22 to − 0.02), with the effect being homogeneous (Q = 7.36, df = 9; P= 0.600). Seven studies reported on the frequency of heavy or binge drinking. Young adults receiving the interventions had a lower frequency of heavy or binge drinking than controls (d = − 0.35; SE, 0.15; 95% CI, − 0.64 to − 0.06), although this measure still showed significant heterogeneity (Q = 29.74, df = 6; P = 0.00004).<br>Adolescents The two studies aimed at adolescent targeted smoking behavior. One study showed a significant difference for whole cigarettes but not for single puff. The other study found no significant differences between a website (6% 30-day point prevalence of abstinence from smoking) and a clinic-based brief office intervention (13%) at 36 weeks.	The web-based interventions in this review reported short-term outcomes, usually 3 months or less, and these may not represent a meaningful change in behavior. Considerable heterogeneity was noted in many of the measures and across the studies. There was considerable diversity in the intensity of the interventions delivered, ranging from an online course to a 15-minute assessment and feedback session. Not all studies provided a control group with an intervention of similar intensity as the experimental intervention.<br>The techniques and process of meta-analysis have received detailed and at times trenchant critiques, with key concerns being the quality of the studies included, combining different measures or interventions, including multiple measures from studies ("inflated *N*s") and publication bias.<br>We did not identify any studies on the effectiveness of web-based interventions with adolescent drinkers.	Cost–benefit analyses comparing web-based with in-person interventions are required. Results of four studies suggest that persistent change in behavior is possible with web-based interventions. Nevertheless, confirmation of long-term impacts is needed.<br>Motivation of adolescents and compliance may be key factors in the effectiveness of school-based of interventions.<br>Studies on the effectiveness of web-based interventions with adolescent drinkers would appear to be an important target for future research, even though it is difficult to obtain the necessary ethics approval to conduct research in this age group, especially when investigating interventions to convert behavior.
MULTIPLE BEHAVIORS			

Study characteristics	*RE-AIM*	*Limitations*	*Recommendations*
Kelders et al. (2012)<br><br>Study type: SR<br><br>Articles: 16/83<br><br>Total sample size: NR<br><br>Target group: NR	[Behaviors]<br>16 interventions targeted a life style behavior, of which 7 weight management, 5 smoking cessation, 1 on nutrition, 1 on physical activity, 1 on both physical activity and nutrition, and 1 on physical activity, nutrition and smoking cessation.<br>[AIM]<br>Overall effect: typical web-based intervention is meant to be used once a week, is modular in setup, is updated once a week, lasts for 10 weeks, includes interaction with the system, a counselor, and peers on the web, includes some persuasive technology elements, and results in about 50% of the participants adhering to the intervention.<br>Intended usage <1/month n=3, 1/month-1/week n=4, 1/week n=6, >1/week n=3.<br>Adherence: mean(SD): 32.8 (23.0)	Non-adherence is an issue in web-based interventions. This frequency of interaction with peers may vary to a large degree between these interventions, but without clear information we cannot make a distinction, which may have influenced our results. It might be that social support has no significant predictive value in this study because of the limited use of social support elements in the included interventions.<br>We have excluded many interventions because data about usage was absent or the usage data that was presented had no direct relationship to the intended use. The description of interventions is varied, which makes it difficult to capture all the characteristics of each intervention.	The description of employed rehearsal in an intervention should be a point of particular interest when (re)designing web-based interventions.<br>The frequency of interaction with a counselor was a significant predictor of adherence. This finding concurs with other reviews that conclude that counselor or clinician support is related to greater exposure and engagement.<br>Reminders and the provision of frequent updates can positively influence adherence. Extensive employment of dialogue support is related to better adherence.<br>When looking at the other persuasive technology categories, we see that social support shows a trend towards a significant contribution to better adherence.<br>Primary task support plays a more important role in the effect of the intervention than in the adherence. Lifestyle interventions, although aimed at long-term goals, might benefit from incorporating segments with a more strict format and shorter duration. Cutting an intervention into shorter segments may be enough to improve adherence, but this should be investigated further. For intervention studies, we would advise researchers to at least provide the information needed (ie, intended usage and usage data related to this intended usage) to calculate this adherence measurement and, preferably, to state the calculated adherence percentage for easy comparison between interventions. Initiatives to standardize and improve the description of web-based interventions like the consort statement for eHealth, a protocol for systematic reviews in eHealth, and guidelines for executing and reporting internet intervention research are therefore very necessary and will hopefully improve the possibility to compare eHealth technologies and learn from each other. It would be interesting to compare interventions that show high adherence with interventions that show low adherence using in-depth, qualitative analyses. It is interesting to test our statistical adherence model in experimental studies. Expanding the model by including the characteristics of participants seems to be relevant. Exploring the relationship between persuasive technology, especially primary task support, and (clinical) outcomes of an intervention is likely to be a worthwhile line of research.
Donkin et al. (2011)<br><br>Study type: SR<br><br>Articles: 16/69	[Behaviors]<br>Five studies targeted at weight management, five at nicotine use, five at physical activity and one fruit and vegetable consumption.<br>[E] Of 69 studies, 33 studies were analyzed for the impact	Strong variation in the definition of adherence, which was found in the reviews studies, is likely to produce mixed findings. Despite having several seemingly objective measures such as number of logins, time spent online, and activities completed, there are still difficulties	Future interventions that intend to measure the impact of adherence on outcomes would benefit from clearly defining their adherence variables and exploring all relationships between the potential adherence variables and outcomes. In addition to this, measures of inactivity need to be included where temporal measures are used.

Study characteristics		*RE-AIM*	*Limitations*	*Recommendations*
Total sample size<br>Target group	12027/19203<br>Adults (18+)	of 1 or more measure of adherence on outcome variables. Of the latter, effectiveness was found in four of five studies on nicotine use, all five studies on weight management, three of five studies on physical activity, the only study on fruit and vegetable intake.<br>[AIM] Mean study discontinuation rate was approximately 23% of all trial participants (range 0%–83%). For participants involved in trials targeting weight changes as an outcome, all measures of adherence were correlated with outcomes. Therefore, more adherent participants had a higher level of weight loss.	in determining the "dose" a participant receives.	Further exploration within the area of e-therapy needs to determine whether adherence influences outcomes through expectation beliefs or through participants being generally adherent to treatment recommendations. To do this, control group adherence behaviors need to be reported more. While objective data can be captured with relative ease, this may not truly reflect the user's experience and dose. User aspects such as processing speed, and familiarity with Web-based platforms and user interfaces are likely to influence the time spent online. Therefore, understanding the contributing factors of adherence is likely to be as important as understanding adherence per se. Program persistence and adherence may be important for physical and psychological interventions in different ways. However, these results need to be considered with caution, given the limitations of this review and the potential biases in the data. We therefore recommend that this be explored in future studies where adherence is the focus. Understanding the differential effects of different measures of adherence will be important in future content and platform development, as well as in evaluating applicability and health service issues.
Brouwer et al. (2011)<br>Study type<br>Articles<br>Total sample size<br>Target group	 SR<br>64<br>NR<br>General public (18+)	[Behaviors]<br>There were 12 studies on physical activity, 4 on nutrition, 14 on weight management, 18 on smoking cessation, 9 on alcohol reduction and 7 on a combination of behaviors.<br>[R] The number of study participants ranged from 32 to 67,324 with an overall mean of 3367 participants and a median of 408. The mean age varied from 32 to 52 years and the percentage of female participants ranging from 2% to 100%. The percentage of participants with education at a level higher than high school (if reported) varied from 41% to 100%.<br>[AIM] The five most used potential exposure improving methods were feedback (used by 73% of interventions), 69% used interactive elements, 67% used email/phone contact, 52% used self-monitoring, and 48% used peer support.<br>Peer support, counselor support, email and/or phone contact with visitors, and updates of the intervention website were related to more exposure. Time spend on the website was positively influenced by peer and counselor support. More average log-ins was related to email/phone contact and updates of the intervention website.<br>There were marked differences in the use of other interactive educational content between the interventions	There was little consistency in the exposure measures that were reported.<br>One possible reason for not finding differences in exposure according to the use of more as compared with fewer interactive behavior change strategies is the way in which we divided the interventions (< 3 or ≥ 3 interactive behavior change strategies) and that we pooled all the interventions targeting different health-related behaviors together.<br>Intervention descriptions were sometimes brief.	It is not only important that objective exposure measures (e.g., starting intervention, completing modules/intervention, frequency of visiting, and duration of visit) are presented in studies evaluating Internet interventions, but it is also important that these measures are presented in a standardized way.<br>For the purpose of systematic reviews, it is very important that accurate and complete descriptions of intervention content and interactive applications are provided in the future. Objective exposure measures should be linked to visitor characteristics to get a more thorough impression about who is reached with what kind of intervention and to what extent. This registration on the individual level would also make it possible to study possible mediating effects of exposure to these objective exposure outcome measures.<br>More research is needed to gain insight into how intervention characteristics can be used to improve exposure to Internet interventions. More accurate and consistent description of intervention content and more consistency in the report of objective exposure outcomes are recommended.

Study characteristics	RE-AIM	Limitations	Recommendations
	for the various target behaviors. Peer support was offered more often in weight management, alcohol and smoking cessation interventions as compared with the other behaviors. Counselor support was more often a distinct part of the weight management and smoking cessation programs.		
Crutzen et al. (2011)<br>Study type: SR<br>Articles: 17<br>Total sample size: NR<br>Target group: Adolescents and young adults	[Behaviors]<br>The 17 reviewed studies focused on smoking cessation (n=6), sexual behavior (n=4), alcohol use (n=2), physical activity (n=2), weight loss (n=1), nutrition (n=1), and healthy lifestyles (n=1).<br>[AIM] Methods found to be used to facilitate exposure were targeted communication, tailored communication, monitoring of health behavior (change), professional and peer support (enabled through discussion board or forum), interactive and easy accessible content, conditional progress (progress depending on finishing previous parts), embedding in a social context (e.g. school-based interventions), use of reminders and incentives.<br>Discussion boards were only moderately used for peer support. Although professional support (e.g. ask-the-expert) was often provided, it was only limitedly used. Providing content in a more interactive way resulted in higher exposure. The combination of tailored communication, the use of reminders to visit or revisit or invitations to participate, and the use of incentives, however, resulted in high exposure to Internet-delivered interventions.	The process of dissemination was described only to a limited extent in the included studies, and most interventions were applied in a research setting and participants were recruited to participate in a study. No exposure- specific theories were mentioned in the reviewed studies. The use of incentives is a strategy that is probably inapplicable once an Internet-delivered intervention is implemented in real-life, because this would increase costs enormously.	It is recommended to conduct experimental research in more controlled settings to increase evidence-based insight into their effectiveness regarding exposure, before applying these strategies in practice. A meta-analytical approach can be used to detect robust effects regarding the effectiveness of certain strategies.<br>In addition to a description of the educational content of an Internet-delivered intervention, future studies should also describe which strategies they have used specifically to attract attention to the intervention and to make using the intervention and revisiting it (when applicable) attractive , to gain more insight into potential effective strategies to improve exposure. More attention should be paid to the development and application of theories regarding exposure, from which methods and strategies can be derived.<br>Effective strategies can be derived from Internet-delivered interventions focusing on different behaviors, but behavior-specific evidence-based insight needs to be gained. With regard to exposure measures, it is recommended to track and report multiple exposure measures. We recommend tracking as many exposure measures as possible because there is no gold standard.<br>Although the focus of this review was not on dissemination of Internet-delivered interventions, successful dissemination is required before participants can be exposed to the intervention.<br>Embedding an Internet/delivered intervention in a social context could be defined as a<br>feasible and appropriate way to disseminate it, for example, by linking the intervention to school activities
Cugelman et al. (2011)<br>Study type: M<br>Articles: 27/30<br>Total sample size: 17,524	[Behaviors]<br>Voluntary lifestyle behaviors (alcohol, smoking, and others).<br>[R] 26 studies reported gender, of which 47.7% of participants were female. 15 reported education level of which the majority (57.6%) had a Bachelor's degree, 23.6% had a Master's degree. 83.7% of participants (19 studies) were White. Mean age ranged from 15.5-66 years (n=26).	Short and tailored interventions can be as effective, if not more effective, than some longer and demanding ones. However, this trend is likely to be limited to particular behaviors, such as responsible drinking and diet choices, but is less applicable to demanding change processes, such as tobacco cessation or weight loss.<br>While coding influence components, some papers only provided vague descriptions, while others did not describe influence components other than those that	Just one intervention reported source credibility even though credibility has been recommended by numerous design guidelines.<br>Too many may potentially overwhelm users with complex and demanding interventions, while there is probably a middle ground where a small number of relevant (and mutually reinforcing) influence components are most effective.<br>The strong and statistically insignificant correlations found in this study suggest that this relationship may require a larger pool of studies to overcome measurement distortions.

Study characteristics		RE-AIM	Limitations	Recommendations
Target group	General public (10+)	Younger audiences achieved the largest behavioral impacts, with impact strength decreasing as participants increased in age.<br>[E] Overall effect size (n=30) was *d*=0.19 CI 0.111-0.278. The overall impact of online interventions is small, with the control conditions explaining much of the variance across studies. This suggests that online intervention efficacy should be regarded as a relative advantage in comparison to different intervention media.<br>Interventions matched against waitlist or placebo control groups achieved the highest effect sizes and contained the largest number of relative influence components (average of 5.7 behavioral determinants and 8.6 behavior change techniques). Interventions compared with website control groups attained a smaller but significant effect size and possessed fewer influence components (average of 4.4 behavioral determinants and 8.3 behavior change techniques). Interventions compared with the sophisticated print intervention control groups were statistically no different from print publications and possessed the fewest influence components (average of 2 behavioral determinants and 3 behavior change techniques).<br>The most effective feedback mechanism was providing feedback on performance. The few interventions that demonstrated similarity to the audience members showed a strong effect size.<br>[AIM] Shorter interventions offer larger impact, while longer interventions offer lower impacts. Interventions that operated longer than 4 months were statistically insignificant, demonstrating no substantial behavioral impact. In general, as the length of an intervention increased, behavioral impacts and intervention adherence decreased.	comprise conventional therapy.<br>Numerous independent calculations will increase the odds of producing false positives.	By better understanding the components of motivation, promoters of healthy lifestyles can potentially design better interventions.
Webb et al. (2010)<br>Study type<br>Articles<br>Total sample size	 M<br>85<br>43,236	[Behaviors]<br>Studies were aimed at physical activity, dietary behavior, alcohol consumption, and smoking abstinence.<br>[E] Interventions were aimed at physical activity, dietary behavior, alcohol consumption, smoking abstinence or a combination of those.<br>Interventions tended to have variable effects on behavior, and the average effect on behavior was statistically small. While some interventions had very large effects (d> 1.00)	Heterogeneity of effects across findings and the relatively small number of interventions associated with some characteristics mean that the findings should be treated with caution.	Future research might usefully consider how particular combinations of techniques might be especially effective in promoting behavior change future research should collect cost-effectiveness data.<br>Relatively few interventions employed stress management and general communication skills training, techniques that were associated with the greatest changes in behavior, so the findings should be treated with caution and form the basis for future research.<br>Descriptive norms can exert a more powerful effect on behavior and decision making than injunctive norms.

Study characteristics		RE-AIM	Limitations	Recommendations
Target group	NR	on behavior, others were found to have small or even negative effects on behavior. Effect sizes were small-to-medium.<br>The findings suggest that the effectiveness of Internet-based interventions is associated with more extensive use of theory (in particular the use of the theory of planned behavior), inclusion of more behavior change techniques, and use of additional methods of interacting with participants (especially text messages).<br>The two behavior change techniques that were associated with the greatest changes in behavior were stress management and general communication skills training. Providing information about others' approval (subjective or injunctive norms) seemed to be less effective than providing normative information about others' behavior (descriptive norms, d+= 0.06 and 0.18, respectively). The effects of modeling on behavior change rendered the effect size significant (k = 4, Q = 13.84, 95% CI = 0.14-0.84, d+= 0.49, P = .006) after the exclusion of one outlier. The use of communicative functions, especially access to an advisor to request advice, also tended to be effective.		It may be that, although the Internet provides a suitable medium for delivering interventions, personal contact via email, online, or text message helps to support behavior change.

[1]Meta-analysis, [2]Proportion eligible studies, [3]Sample sizes corresponding to (eligible) studies, [4]Systematic review, [5]Not reported
